# Kinetic inductance of few-layer NbSe_2_ in the two-dimensional limit

**DOI:** 10.1038/s41467-026-75672-8

**Published:** 2026-07-23

**Authors:** Sameia Zaman, Joel Î-j. Wang, Thomas Werkmeister, Miuko Tanaka, Thao Dinh, Max Hays, Daniel Rodan-Legrain, Aranya Goswami, Réouven Assouly, Ahmet Kemal Demir, David K. Kim, Bethany M. Niedzielski, Kyle Serniak, Mollie E. Schwartz, Kenji Watanabe, Takashi Taniguchi, Philip Kim, Riccardo Comin, Jeffrey A. Grover, Terry P. Orlando, Pablo Jarillo-Herrero, William D. Oliver

**Affiliations:** 1 Research Laboratory of Electronics, Massachusetts Institute of Technology, Cambridge, MA USA; 2 Department of Electrical Engineering and Computer Science, Massachusetts Institute of Technology, Cambridge, MA USA; 3 Department of Physics, New York University, New York, NY USA; 4 John A. Paulson School of Engineering and Applied Sciences, Harvard University, Cambridge, MA USA; 5 Department of Physics, Massachusetts Institute of Technology, Cambridge, MA USA; 6 Lincoln Laboratory, Massachusetts Institute of Technology, Lexington, MA USA; 7 Research Center for Electronic and Optical Materials, National Institute for Materials Science, Tsukuba, Japan; 8 Research Center for Materials Nanoarchitectonics, National Institute for Materials Science, Tsukuba, Japan; 9 Department of Physics, Harvard University, Cambridge, MA USA

**Keywords:** Superconducting properties and materials, Superconducting devices, Two-dimensional materials

## Abstract

Van der Waals (vdW) superconductors remain superconducting down to the monolayer limit, enabling the exploration of emergent physical phenomena and functionality driven by reduced dimensionality. Here, we report the characterization of the kinetic inductance of atomically thin NbSe_2_, a two-dimensional van der Waals superconductor, using superconducting coplanar waveguides and microwave measurement techniques familiar to circuit quantum electrodynamics (cQED). The kinetic inductance scales inversely with the number of NbSe_2_ layers, reaching 1.2 nH/***□*** in the monolayer limit. Furthermore, the measured kinetic inductance exhibits a thickness-dependent crossover from clean- to dirty-limit behavior, with enhanced dirty-limit contributions emerging in the ultra-thin regime. These effects are likely driven by increased surface scattering, multi-band superconductivity, and geometric confinement. Additionally, the self-Kerr nonlinearity of the NbSe_2_ films ranges from ***K/2π*** = −0.006 to −14.7 Hz/photon, indicating its strong potential in applications requiring compact, nearly linear, high-inductance superconducting quantum devices and detectors. The fabrication and characterization techniques demonstrated here are extensible to the investigation of other two-dimensional superconductors.

## Abstract (web summary)

The authors study the kinetic inductance of hBN-encapsulated superconducting 2H-NbSe_2_ thin films using microwave techniques. The kinetic inductance scales inversely with the number of layers and exhibits a thickness-dependent crossover from clean- to dirty-limit behavior, with enhanced dirty limit contributions in the ultra-thin regime.

## Introduction

Kinetic inductance (*L*_k_) in a superconductor arises from the inertia of Cooper pair transport in response to a varying electromagnetic field. Unlike geometric inductance, which varies with the geometry of the electronic element, kinetic inductance depends on intrinsic properties of the material, e.g., superfluid density, carrier effective mass, and film thickness. For example, in a clean superconductor, the kinetic inductance per square is *L*_k,sq_ = *m*_eff_/*e*^2^*n*_0_*d*, where *m*_eff_ is the effective mass of the charge carriers, *n*_0_ is the superfluid density (number of Cooper pairs per unit volume), and *d* is the thickness of the sample. This relation indicates that *L*_k,sq_ increases with decreasing film thickness or suppressed superfluid density. Such an enhancement is useful in quantum circuits, where superconductors with high-*L*_k_ are used to realize superinductors—inductive elements with a reactance that approaches and even exceeds the quantum unit *R*_Q_ = *h*/(2*e*)^2^ = 6.4 kΩ for Cooper pairs, where *h* is the Planck constant, and *e* is the elementary charge. Furthermore, the kinetic inductance in a superconductor is directly related to the superfluid stiffness; probing it enables insight into several fundamental properties such as the superconducting gap structure and pairing symmetry in unconventional superconductors^1–6^.

Superinductors are used in a variety of applications, including in superconducting qubit circuits such as fluxonium and 0–*π* qubits, which require large (and ideally linear) shunt inductance^7,8^. They have also been used to realize coherent quantum phase-slip circuits^9,10^, long-range coupling for spin qubits^11^, and tailored Kerr non-linearities in light–matter interactions^12,13^. Furthermore, these materials can be used for wide-band parametric amplifiers and high-impedance readout resonators^14–18^. For many of these applications, low-*L*_k_ aluminum is used, and thus, large inductance is realized primarily by an array of Al–AlO_*x*_–Al Josephson junctions^19–21^. However, these junctions exhibit strong nonlinearity and are therefore limited in the current they can support while remaining in the linear inductance regime. This motivates the search for alternative materials that can provide large inductance with minimal nonlinearity. Recent efforts toward this goal have focused on implementing superinductors using amorphous superconducting thin films, such as TiN^22,23^, NbN^24,25^, NbTiN^26,27^, InO_*x*_^9,28^, NbSi^29^, WSi^30^, AlO_*x*_^31^, and granular Al^32,33^. The inherent disorder of these materials suppresses the density of Cooper pairs, leading to high-*L*_k_ in the superconducting regime^34^. Typically, these amorphous superconductors operate in the dirty limit, where the mean free path of charge carriers is shorter than the superconducting coherence length^35^. This relationship implies that, in the BCS framework, the size of a Cooper pair is greater than the average travel distance between consecutive scattering sites. In contrast, the coherence length of a clean superconductor is shorter than its mean free path, and Cooper pair transport can be regarded as ballistic when the length of the device is shorter than the mean free path. In superconducting quantum devices, disorder and the resulting scattering of charge carriers are expected to affect quasiparticle dynamics and, consequently, device coherence. While the link between disorder, quasiparticle generation, and the associated noise remains an area of active investigation^32,36–38^, microwave characterization of superconductors with varied disorder levels may offer a way to probe their dynamic responses and help explore the role of scattering in decoherence^38^.

**Fig. 1:**
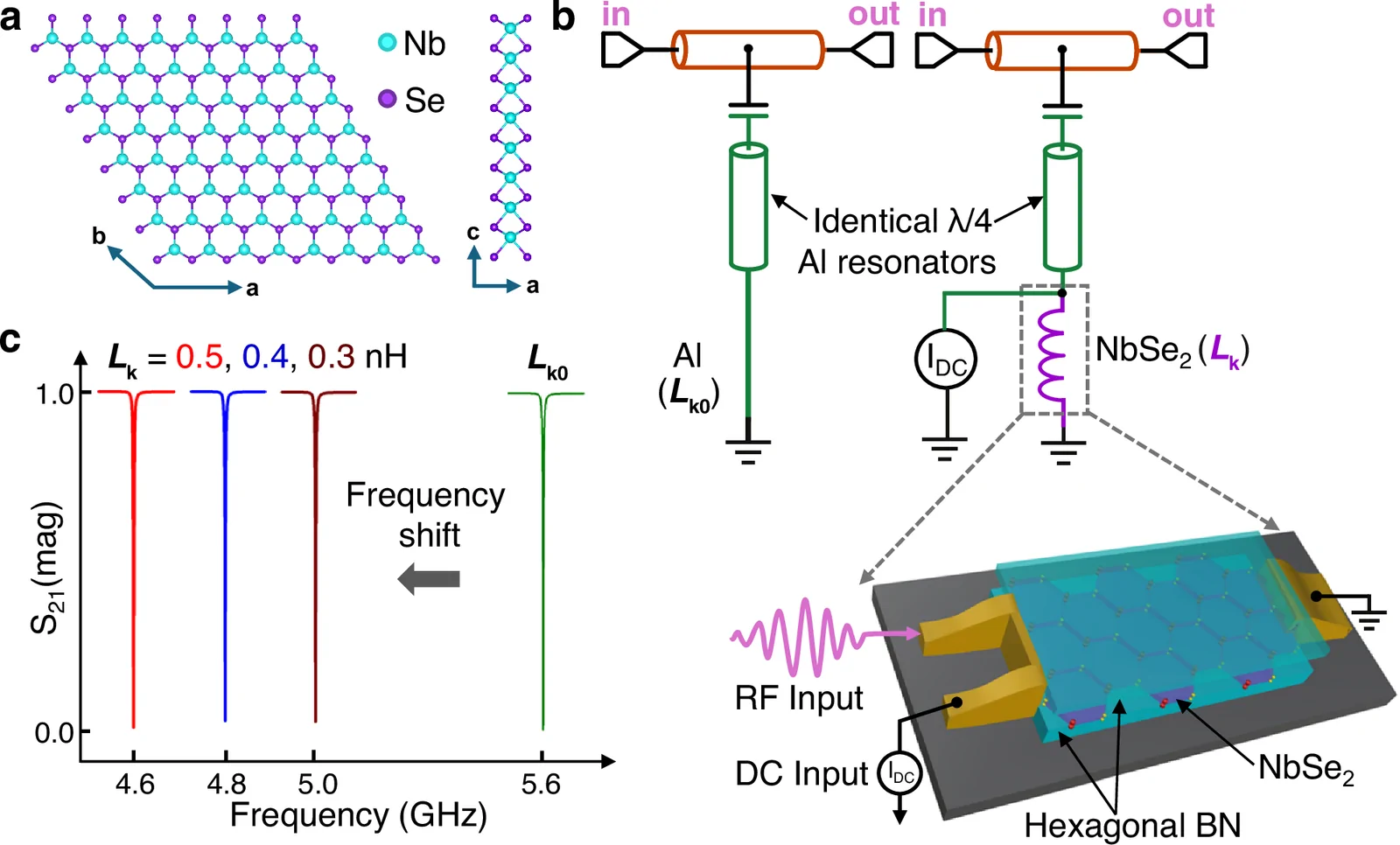
Superconducting resonators and terminations to characterize the kinetic inductance of NbSe_2_. **a** Crystal structure of monolayer 2H-NbSe_2_, top-down and side views. **b** Measurement circuit schematic. CPW resonators are capacitively coupled to a common transmission line and terminated either directly to ground via aluminum (control) or through a hBN–NbSe_2_–hBN heterostructure (experiment). The resonant frequency *f*_r_ of the aluminum *λ*/4-resonator is determined by the effective inductance and capacitance of the CPW. Termination by NbSe_2_ introduces additional kinetic inductance, shifting the resonant frequency to a new value $f_{r}^{\prime}$. (Zoom-in of inductor) hBN–NbSe_2_–hBN heterostructure contacted by Al electrodes with both RF and DC input signals applied across the sample and terminated at ground. **c** Simulated resonator spectra illustrating the effect of increased inductance on the resonant frequency. *L*_k0_ denotes the inductance of the aluminum-terminated resonator, while *L*_k_ represents the additional inductance introduced by the NbSe_2_ sample.

We investigate 2H-NbSe_2_ (further referred to as NbSe_2_ for brevity), a transition-metal dichalcogenide and a type-II superconductor. The lattice structure of NbSe_2_ is hexagonal, with the Nb atoms in trigonal prismatic coordination surrounded by Se atoms (Fig. 1a). The layers are stacked in an ABAB sequence along the *c*-axis and are bonded by van der Waals forces between adjacent layers. NbSe_2_ possesses out-of-plane mirror symmetry and broken in-plane inversion symmetry. This broken in-plane symmetry gives rise to an unconventional Ising spin–orbit coupling, which maintains superconductivity in the presence of a strong in-plane magnetic field exceeding 30 T, as observed in the monolayer limit^39,40^. Both superconductivity and the charge–density wave transition exist from the bulk down to monolayer thickness. However, the superconducting transition temperature (*T*_c_) decreases with reduced film thickness, attributable to a suppressed Cooper-pair density, the presence of a superconductor–vacuum interface, and modifications to the electronic band structure^41,42^. As the thickness is reduced, NbSe_2_ exhibits several unusual quantum phenomena, including thickness-dependent Higgs modes^43^, dissipationless phase diagrams^44^, unconventional finite momentum pairing^45–47^, and the emergence of a quantum metallic phase under a small perpendicular magnetic field^48^. Prior direct current (DC) transport studies indicate that the carrier mean free path in NbSe_2_ decreases with its thickness due to enhanced surface scattering^41,44^. When encapsulated by hBN, even bilayer and monolayer NbSe_2_ exhibit mean free paths (approximately 17 and 15 nm, respectively) that are larger than their corresponding superconducting coherence length (8 nm)^40,41,45^. These DC transport measurements are consistent with encapsulated NbSe_2_ exhibiting superconductivity in the clean limit^41,49–51^.

In this work, we measure the kinetic inductance to access the complex conductivity of few-layer NbSe_2_ in the two-dimensional limit. This method allows us to determine whether the material lies in the clean limit, the dirty limit, or an intermediate regime. Moreover, it is sensitive to fundamental superconducting properties such as superfluid stiffness, surface impedance, and loss mechanisms^4,52,53^, providing insight into the material suitability for superconducting quantum devices operating in the microwave regime.

## Results

We study the kinetic inductance of hBN-encapsulated NbSe_2_ thin films using quarter-wavelength *λ*/4 coplanar waveguide (CPW) resonators made of thin-film Al. The resonant frequency of a resonator is ${f}_{{{{\rm{r}}}}}=\frac{1}{2\pi \sqrt{{L}_{{{{\rm{eff}}}}}{C}_{{{{\rm{eff}}}}}}}$, where *L*_eff_ and *C*_eff_ are the effective inductance and capacitance of the resonator, respectively (Fig. 1b, left panel). The Al inductance is primarily geometric, and its kinetic inductance per square is much smaller than that of NbSe_2_. When the *λ*/4-resonator is terminated to ground by an NbSe_2_ sample, the resonant frequency shifts to a lower frequency $f_{r}^{\prime}=\frac{1}{2\pi\sqrt{L_{{\mathrm{eff}}}^{\prime}C_{{\mathrm{eff}}}^{\prime}}}$ due to the additional kinetic inductance from the NbSe_2_ (Fig. 1b, right panel). We perform microwave simulations to determine the expected frequency shift of a *λ*/4-resonator due to the additional inductance *L*_k_ (Fig. 1c). The Al-only resonator has a frequency of 5.6 GHz. When terminated by NbSe_2_, the resonance frequency decreases by 200 MHz per 0.1 nH of added termination inductance.

**Fig. 2:**
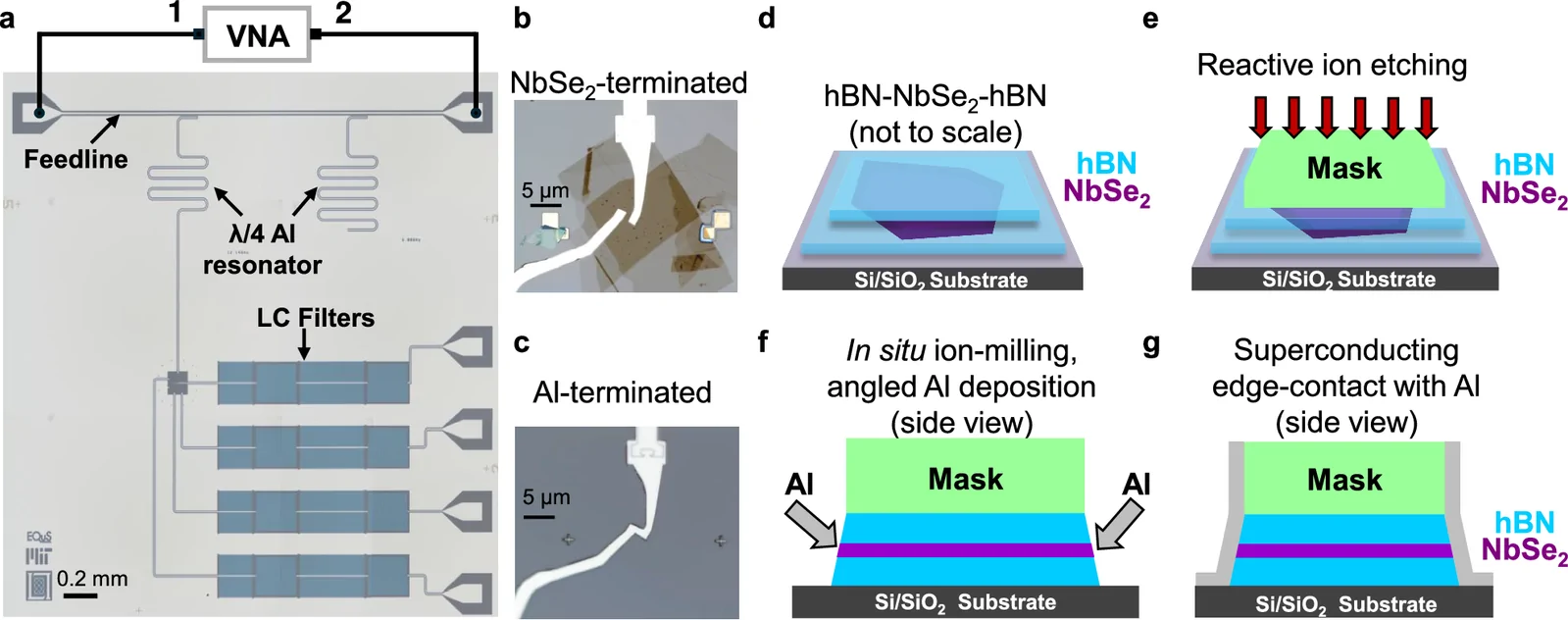
Measurement configuration and device fabrication for the kinetic inductance measurement of NbSe_2_. **a** Optical micrograph of a 5 × 5 mm^2^ chip containing CPW resonators, a shared feedline, DC bias lines, on-chip LC filters, and a ground plane, all patterned from 250 nm-thick aluminum on a high-resistivity silicon substrate. Only one (of four) DC bias lines is used in this experiment. **b** and **c** Optical micrographs of the NbSe_2_-terminated and aluminum-terminated *λ*/4 CPW resonators, respectively. **d–g** Illustration of the edge-contact fabrication process. The NbSe_2_ (purple) flake is fully encapsulated by hBN (blue) and patterned via reactive-ion etching (RIE) to expose its edges. After in-situ argon ion milling, superconducting edge contacts are formed by angled aluminum evaporation with substrate rotation.

Figure 2a shows an optical micrograph of the superconducting circuit with *λ*/4 CPW resonators used for this work. The aluminum CPW resonators are fabricated from a 250 nm Al film deposited on a high-resistivity silicon substrate and patterned using a combination of photolithography and wet etching (see ref. ^4^ and the “Methods” section for details). Two *λ*/4-resonators, an aluminum spectator resonator, and an experiment resonator, are capacitively coupled to a common feedline. The spectator resonator is used as a witness to monitor the impact of the vdW materials fabrication process (without incorporating vdW materials), while the experiment resonator is terminated by a NbSe_2_ sample situated within a square hosting window of size 200 μm × 200 μm. To extract the kinetic inductance of each NbSe_2_ device, we analyze two *λ*/4-resonators: the NbSe_2_-terminated experiment resonator (Fig. 2b) and a control resonator of the same geometry that is terminated instead by a 250 nm-thick aluminum film (Fig. 2c). The NbSe_2_ thin film is encapsulated in hBN to maintain a nominally clean and atomically flat interface while ensuring a relatively low-microwave-loss environment^53,54^. This hBN–NbSe_2_–hBN heterostructure is assembled through mechanical exfoliation and standard dry-transfer techniques inside an argon-filled glove box, ensuring minimal oxidation of the NbSe_2_ before full encapsulation.

The hBN–NbSe_2_–hBN heterostructure is galvanically connected to the microwave resonator and the ground plane. To achieve a highly transparent, superconducting contact, we make a one-dimensional edge-contact to the NbSe_2_ using reactive ion etching of the heterostructure, followed by in-situ argon ion milling and aluminum deposition (Fig. 2d)^55^. A low-loss superconducting contact is critical to maintaining a narrow resonance line (i.e., a high quality factor of the resonator), which translates to higher measurement resolution of the resonator frequency. Details of the fabrication process and the verification of the superconducting contact are provided in the Supplementary Note 1 and Supplementary Table 1.

**Fig. 3:**
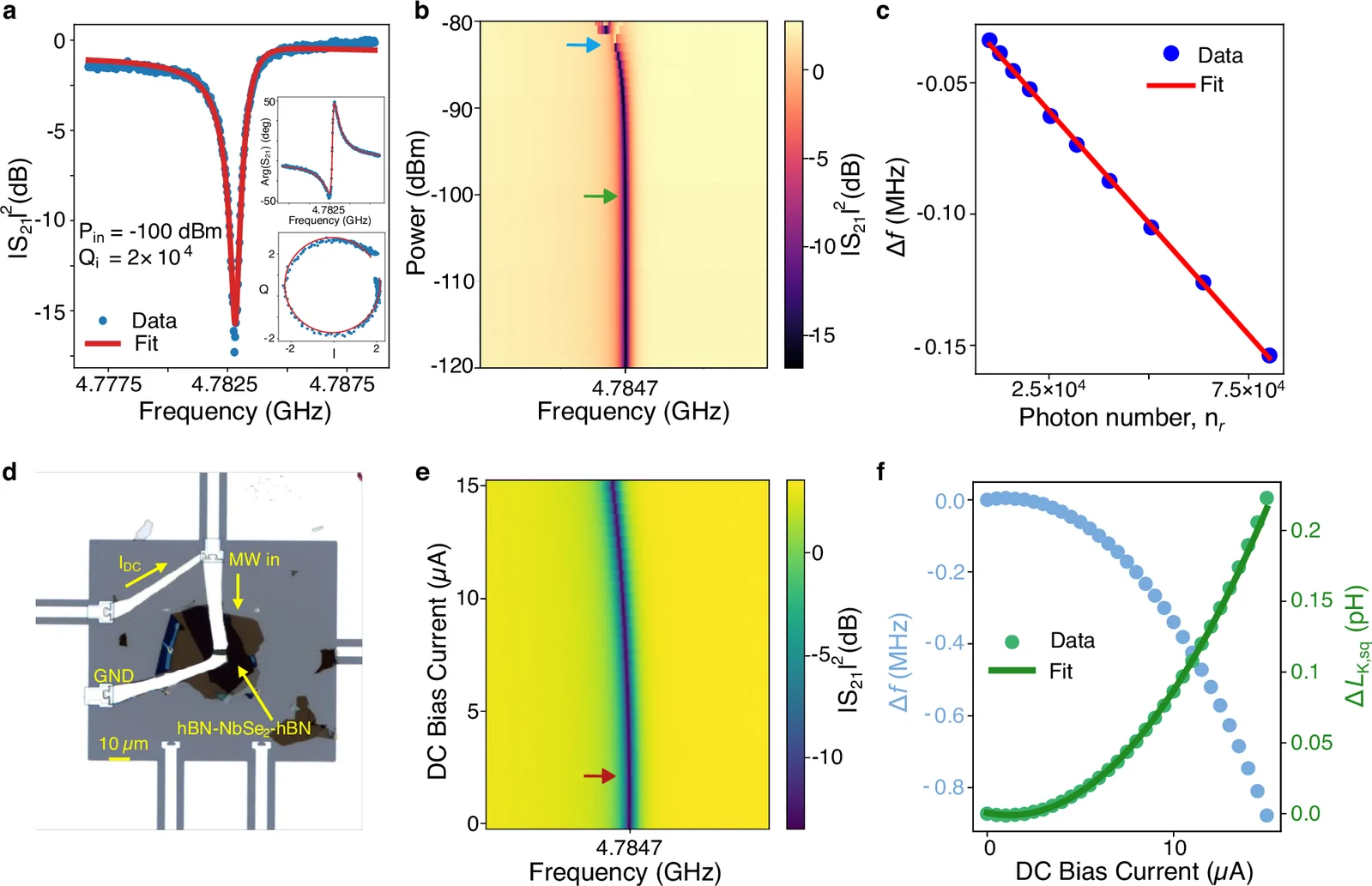
Microwave measurements of NbSe_2_-terminated *λ*/4-resonator. **a** Transmission coefficient (∣*S*_21_∣^2^) of a NbSe_2_-terminated *λ*/4-resonator measured at 10 mK (Device ID D6). Inset: Complex I-Q plane showing the measured response (blue) and Lorentzian fit (red). **b** Transmission coefficient (∣*S*_21_∣^2^) of NbSe_2_-terminated *λ*/4-resonator as a function of input microwave power (*P*_rf_). The resonant frequency (${f}_{{{{{\rm{r,Al-NbSe}}}}}_{{{{\rm{2}}}}}}$) shifts to the lower frequency with increasing microwave power. The onset of the frequency shift—defined as exceeding one standard deviation from the low-power baseline (${f}_{{{{{\rm{r,Al-NbSe}}}}}_{{{{\rm{2}}}}}}=$ 4.785 GHz)—is observed at an input power of −100 dBm (indicated by the green arrow). At −82 dBm, the resonant peak bifurcates (blue arrow), indicating the onset of a bistable regime. **c** Linear fit of the resonant frequency shift $(\Delta f={f}_{{{{{\rm{r,Al-NbSe}}}}}_{{{{\rm{2}}}}}}({n}_{{{{\rm{r}}}}})-{f}_{{{{{\rm{r,Al-NbSe}}}}}_{{{{\rm{2}}}}}}({n}_{{{{\rm{r}}}}}=1))$ as a function of resonator photon number (*n*_r_) in the NbSe_2_-terminated *λ*/4-resonator. **d** Optical image of the NbSe_2_-terminated *λ*/4-resonator with a DC bias line connected at the microwave input port. **e** DC bias dependence of the NbSe_2_-terminated *λ*/4-resonator measured at a fixed microwave power of *P*_rf_ = −120 dBm. The resonant frequency shifts to the lower frequency with increasing bias current. The onset of the frequency shift—defined as exceeding one standard deviation from the baseline (${f}_{{{{{\rm{r,Al-NbSe}}}}}_{{{{\rm{2}}}}}}=$ 4.785 GHz)—is observed at a bias current of 1 μA (maroon arrow). **f** Extracted resonant frequency shift ($\Delta f={f}_{{{{{\rm{r,Al-NbSe}}}}}_{{{{\rm{2}}}}}}({I}_{{{{\rm{DC}}}}})-{f}_{{{{{\rm{r,Al-NbSe}}}}}_{{{{\rm{2}}}}}}({I}_{{{{\rm{DC}}}}}=0)$) and kinetic inductance shift ($| \Delta L_{{\rm{k,sq}}} | = L_{{\rm{k,sq}}} (I_{{\rm{DC}}}) = 0$) *L*_k,sq_(*I*_DC_)) of the NbSe_2_-terminated resonator as a function of DC bias current. Measured data (circle-green and sky-blue) and theoretical fit (line-green) are shown.

The samples are characterized in a dilution refrigerator with a base temperature of ~10 mK (see Supplementary Fig. 2 for wiring diagram). The microwave transmission coefficient *S*_21_ is measured as a function of frequency using a two-port vector network analyzer (VNA). Fig. 3a shows a representative ∣*S*_21_∣ measurement from a *λ*/4-resonator terminated by a 9 nm thick (determined by atomic force microscopy) NbSe_2_ film (Device ID D6, see Table 1). The resonant frequency ${f}_{{{{{\rm{r,Al-NbSe}}}}}_{{{{\rm{2}}}}}}$ and quality factor are determined from a Lorentzian fit using the standard circular fit procedure^56^ (Fig. 3a inset and Supplementary Note 2). In the single-photon limit, the NbSe_2_-terminated resonator exhibits a resonant frequency ${f}_{{{{{\rm{r,Al-NbSe}}}}}_{{{{\rm{2}}}}}}$ = 4.79 GHz and an internal quality factor ${Q}_{{{{{\rm{i,Al-NbSe}}}}}_{{{{\rm{2}}}}}}=2.0\times 1{0}^{4}$, while the Al-terminated control resonator has a resonant frequency *f*_r,Al_ = 5.34 GHz and an internal quality factor *Q*_i,Al_ = 1.0 × 10^5^. The lower internal quality factor observed in the NbSe_2_-terminated Al resonator is likely due to additional dissipation arising from imperfect electrical contacts between the Al and the NbSe_2_, as well as dielectric loss introduced by residual polymer contamination from the dry transfer process. Moreover, the frequency difference ${f}_{{{{\rm{r,Al}}}}}-{f}_{{{{{\rm{r,Al-NbSe}}}}}_{{{{\rm{2}}}}}}$ = 0.55 GHz highlights the impact of terminating the resonator with NbSe_2_ and enables us to extract a kinetic inductance *L*_k,sq_ = 124 pH/□ for this NbSe_2_ sample.

**Table 1:** Summary of kinetic microwave characterization of NbSe_2_ samples across different thicknesses

Device ID	*d* (nm)	*W* (μm)	*L* (μm)	$f_{{{\rm{r,Al-NbSe}}}_{2}}$ (GHz)	*f*_r,Al_ (GHz)	*L*_k_ (nH)	*L*_k,sq_ (nH/sq)	$Q_{{{\rm{i,Al-NbSe}}}_{2}}$	*K*/2*π* (Hz)
D1	1.99	3.1	3.1	3.62	5.30	1.195	1.195	0.8 × 10^4^	−14.7
D2	4.08	2.5	5.7	4.14	5.21	0.643	0.282	6.0 × 10^4^	−3.8
D3	5.86	2.6	3.0	4.88	5.33	0.220	0.191	0.7 × 10^4^	−2.9
D4	7.13	4.8	6.7	4.93	5.35	0.200	0.143	2.4 × 10^4^	−2.6
D5	7.98	4.5	12.0	4.63	5.33	0.360	0.135	1.2 × 10^4^	−1.9
D6	8.81	3.9	8.6	4.79	5.34	0.274	0.124	2.0 × 10^4^	−1.7
D7	8.90	4.5	4.2	5.26	5.49	0.100	0.107	2.1 × 10^4^	−1.5
D8	10.07	2.5	6.6	5.16	5.49	0.149	0.056	0.7 × 10^4^	−0.4
D9	11.89	6.0	6.9	5.46	5.60	0.057	0.049	0.6 × 10^4^	−0.03
D10	24.73	4.1	7.5	5.34	5.37	0.014	0.008	18 × 10^4^	−0.006
D11	30.20	3.4	6.1	5.42	5.44	0.009	0.005	2.5 × 10^4^	−0.006
D12	52.12	2.3	4.6	5.6	5.62	0.008	0.004	5.8 × 10^4^	−0.008

*W* and *L* represent the nominal width and nominal length of the NbSe_2_ samples with a thickness of *d*. ${{Q}}_{{{{{\rm{i,Al-NbSe}}}}}_{{{{\rm{2}}}}}}$ and Kerr coefficients (*K*/2*π*) are extracted from NbSe_2_-terminated Al resonators. The film thickness *d* is measured with atomic force microscopy.

The resonant frequency of the NbSe_2_-terminated resonator decreases with increasing microwave probe power, e.g., with a noticeable downshift in ${f}_{{{{{\rm{r,Al-NbSe}}}}}_{{{{\rm{2}}}}}}$ at the estimated sample power of −90 dBm (Fig. 3b, green arrow). This frequency downshift indicates a contribution from kinetic inductance. As the microwave power increases, enhanced Cooper-pair breaking leads to a reduction in the superfluid density (*n*_0_), resulting in a larger kinetic inductance *L*_k_ and, consequently, a lower resonant frequency. In contrast, the aluminum resonator exhibits no observable power dependence up to an estimated input power of −60 dBm at the sample (see Supplementary Fig. 4), corresponding to the upper limit of our VNA output (VNA outputs a power of 10 dBm at room temperature, which is reduced to −60 dBm at the sample after passing through a total attenuation of 70 dB), confirming that its inductance is predominantly geometric. The nonlinear microwave power dependence exhibited by the NbSe_2_-terminated resonator, observed over the power range from −100 to −80 dBm, is well-described by a Kerr-type or Duffing-type nonlinearity^57^ (see Supplementary Note 3). When the microwave power in the resonator exceeds a critical threshold, the resonant frequency bifurcates and enters a bistability regime (Fig. 3b, blue arrow)^58–60^. From a linear fit of the resonant frequency shift ($\Delta f={f}_{{{{{\rm{r,Al-NbSe}}}}}_{{{{\rm{2}}}}}}({n}_{{{{\rm{r}}}}})-{f}_{{{{{\rm{r,Al-NbSe}}}}}_{{{{\rm{2}}}}}}({n}_{{{{\rm{r}}}}}=1)$) with the photon number of the resonator (*n*_r_), shown in Fig. 3c, we extract a self-Kerr coefficient *K*/2*π* = −1.7 Hz/photon at 10 mK. Typically, conventional superconducting inductors of similar dimensions, such as granular aluminum, with a self-Kerr coefficient of 4.5 MHz^61^, and Josephson junction arrays, with a self-Kerr of 8 MHz^62^–exhibit significantly higher nonlinearity. In contrast, NbSe_2_ films show a lower self-Kerr coefficient, indicating low nonlinearity.

We also investigate the dependence of the kinetic inductance on the DC bias current. A DC bias current *I*_DC_ is introduced via a contact to the microwave line just above the NbSe_2_ termination (Fig. 3d), while a microwave tone is maintained in the low-power linear regime (*P*_rf_ = −120 dBm). The resonant frequency (${f}_{{{{{\rm{r,Al-NbSe}}}}}_{{{{\rm{2}}}}}}$) decreases with an increasing bias current (up to *I*_DC_ = 15 μA), indicating a corresponding increase in kinetic inductance (Fig. 3e). This behavior is similar to the microwave response due to the Kerr-type nonlinearity. In the Ginzburg–Landau framework, the dependence of kinetic inductance of the superconducting film with bias current is well-described by a quadratic function (ignoring the terms beyond 2nd order)^35^:

1 $$\begin{array}{r}{L}_{{{{\rm{k}}}}}({I}_{{{{\rm{DC}}}}})={L}_{{{{\rm{k}}}}}(0)\left[1+{\left(\frac{{I}_{{{{\rm{DC}}}}}}{{I}^{*}}\right)}^{2}+...\right],\end{array}$$

where *L*_k_(0) is the kinetic inductance of the resonator at zero current bias, and I^*^ is a characteristic current, also known as the depairing current. I^*^ is typically comparable to the superconducting critical current *I*_c_, and sets the scale of non-linearity. By fitting to this relationship, I^*^ is determined to be 0.4 mA for the 9 nm thick, 3.9 μm wide NbSe_2_ sample. We observe that both the resonant frequency and the kinetic inductance per square exhibit a quadratic dependence on the DC bias current (Fig. 3f), consistent with the fully gapped pairing of NbSe_2_ reported in previous studies^46,63^.

## Discussion

**Fig. 4:**
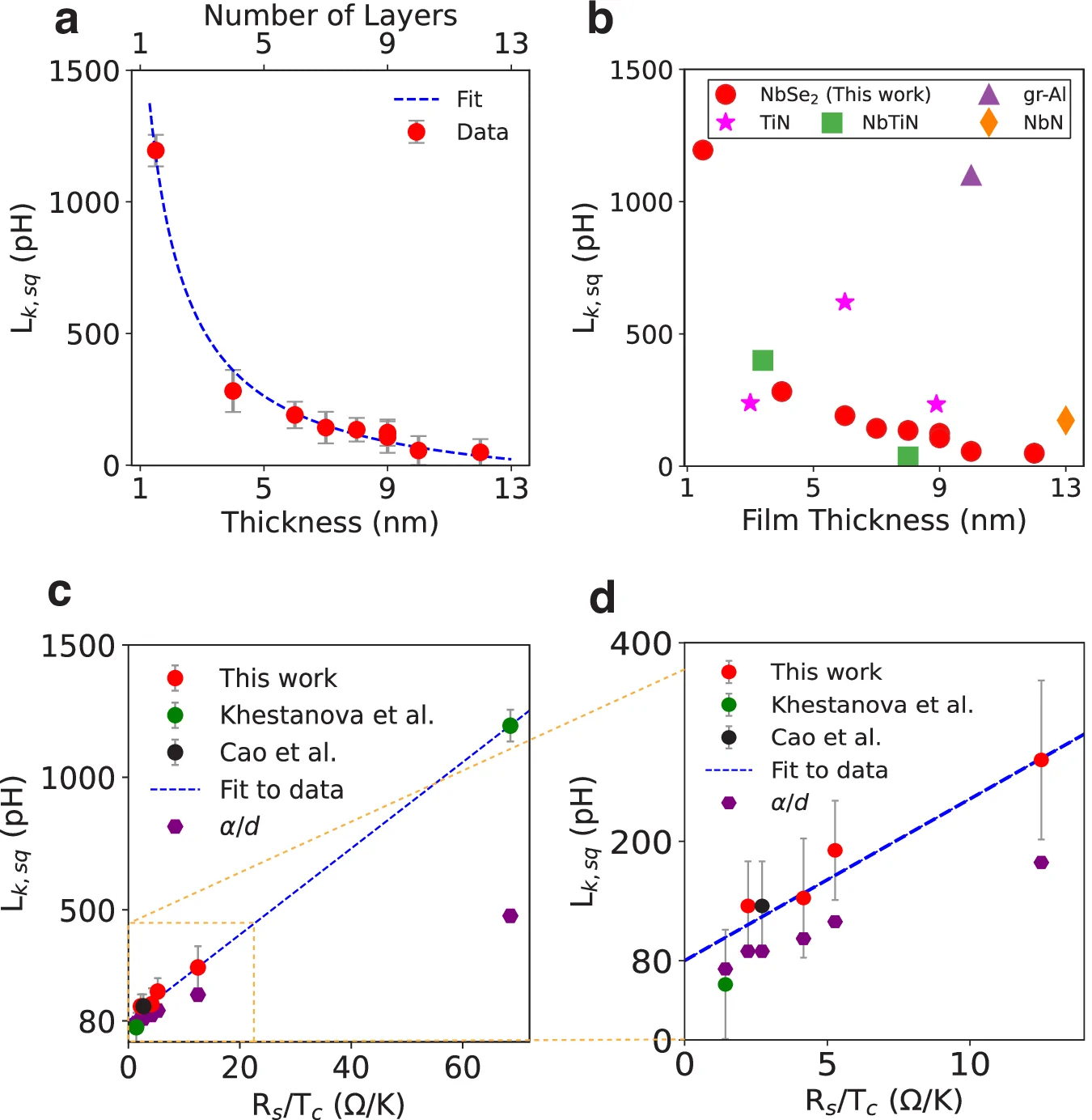
Thickness dependence of kinetic inductance in NbSe_2_. **a** Sheet kinetic inductance (*L*_k,sq_) from microwave measurements (circle-red) of nine NbSe_2_ devices with varying film thicknesses. The blue dashed line shows a 1/*d* scaling fit. **b** Comparison of measured *L*_k,sq_ in NbSe_2_ with values reported for other high-impedance intrinsic superconducting thin films^22,24,26,27,61,65^. **c** Sheet kinetic inductance (*L*_k,sq_) from microwave measurements is plotted as a function of *R*_s_/*T*_c_, where *R*_s_ and *T*_c_ values are obtained from both our transport measurements (red circles) and previous studies (green and black circles)^41,42^. The blue dashed line is a linear fit with a slope of 16.3 and a *y*-intercept of 80.0 pH. The clean-limit contribution ($\alpha /d=\frac{{m}_{{{{\rm{eff}}}}}}{{n}_{0}{e}^{2}d}$) of total kinetic inductance for each data point is plotted (purple hexagons) using an estimate of *α*. **d** The magnified view of the lower region for clarity. The error bars in the plots include contributions from both geometric uncertainty of the device and the uncertainty in the resonance frequencies extracted from circle fits to the complex *S*_21_ data.

VdW superconductors remain superconducting in the monolayer limit^39,64^, which enables one to increase kinetic inductance by reducing the thickness. Figure 4a shows the sheet kinetic inductance (*L*_k,sq_) of NbSe_2_ films extracted from microwave measurements as a function of thickness (*d*) and the corresponding layer numbers. The measured *L*_k,sq_ (red circles) exhibits an approximate 1/*d* dependence, as expected for thin superconducting films, and reaches 1.2 nH for the monolayer sample (see Supplementary Note 6 for the determination of layer numbers). The values are compared to those of other intrinsic superinductors based on amorphous materials, including TiN^22,65^, NbTiN^26,27^, granular aluminum (gr-Al)^61^, and NbN^24^ across a range of film thicknesses up to 13 nm, as shown in Fig. 4b. Voltage-induced flat-band superconductors, such as magic-angle twisted bilayer^4,66^ and trilayer graphene^5,67^, exhibit a wide range of inductances depending on bias voltages and are therefore not included in Fig. 4b.

The observed layer dependence may provide insight into the underlying mechanisms responsible for the high *L*_k_, including the role of scattering processes, and whether our NbSe_2_ devices operate in the dirty limit, the clean limit, or an intermediate regime. We consider a general expression for the sheet kinetic inductance *L*_k,sq_ that spans from the clean to the dirty limit (see Supplementary Note 4 for the derivation):

2 $${L}_{{{{\rm{k,sq}}}}}=\frac{1}{d}\left(\frac{{m}_{{{{\rm{eff}}}}}}{{n}_{0}{e}^{2}}+\frac{\hslash }{1.764 \pi {k}_{{{{\rm{B}}}}}}\frac{{\rho }_{{{{\rm{s}}}}}}{{T}_{{{{\rm{c}}}}}}\right)=\frac{{m}_{{{{\rm{eff}}}}}}{{n}_{0}{e}^{2}d}+\frac{\hslash }{1.764 \pi {k}_{{{{\rm{B}}}}}}\frac{{R}_{{{{\rm{s}}}}}}{{T}_{{{{\rm{c}}}}}}=\frac{\alpha }{d}+\beta \frac{{R}_{{{{\rm{s}}}}}}{{T}_{{{{\rm{c}}}}}}$$

Here, *ρ*_s_ denotes the normal-state resistivity, *ℏ* is the reduced Planck’s constant, *k*_B_ is the Boltzmann constant, and *R*_s_ is the normal-state sheet resistance. If we assume that the term in parentheses varies weakly with film thickness, we expect the approximate 1/*d* dependence observed in Fig. 4a. This expression suggests that the total kinetic inductance arises from a combination of clean-limit (first term) and dirty-limit (second term) contributions. Figure 4c plots the measured sheet kinetic inductance *L*_k,sq_ and clean-limit contribution (the first term of Eq. (2)) as a function of *R*_s_/*T*_c_. The *R*_s_ and *T*_c_ values are obtained from both our transport measurements and previous studies^41,42^ (shown in green and black circles in Fig. 4c and summarized in Supplementary Table 3). We estimate *α* $\left(=\frac{{m}_{{{{\rm{eff}}}}}}{{n}_{0}{e}^{2}}\right)$ for the clean-limit contribution, assuming *α* is independent of disorder and film thickness (see Supplementary Fig. 5). We observe a linear dependence for the larger values of *L*_k,sq_ in Fig. 4c. A linear fit yields a slope *β* = 16.3, which captures the dirty-limit contribution. In contrast, the *α*/*d* term for each data point reflects the clean-limit component, which approaches the total *L*_k,sq_ in thicker samples. As the sample thickness approaches the monolayer limit, the ratio of the measured kinetic inductance to the clean-limit kinetic inductance (*L*_k,sq_/*L*_k,sq,clean_) increases from 0.7 to 15 monotonically, indicating that the dirty-limit contribution becomes increasingly significant in thinner NbSe_2_ samples. This model captures the crossover from clean to the dirty-limit behavior as the sample thickness decreases.

We now discuss possible origins of the observed crossover behavior. In thicker NbSe_2_ films, the majority of charge carriers reside in the interior layers of the 2D crystal. The clean-limit-like behavior observed in this regime—corresponding to the lower-left corner of Fig. 4c—may be attributed to the high crystallinity of the bulk material and effective screening from carriers in the top and bottom NbSe_2_ layers. In contrast, thinner devices exhibit a greater influence from their surrounding environment. In the monolayer limit, all charge carriers are in close proximity to the encapsulating hBN on both sides, leading to enhanced surface scattering from impurities or fabrication residues. This increased scattering may result in more pronounced dirty-limit contributions to the kinetic inductance. Moreover, NbSe_2_ has been shown to host two superconducting bands with distinct gap sizes, with the larger gap decreasing monotonically as thickness is reduced^63^. In other multi-band superconductors^68,69^, such disparity across the Fermi surface has been linked to the coexistence of clean- and dirty-limit behaviors, arising from band-dependent coherence lengths and scattering rates. Our experiment does not explicitly resolve contributions from individual bands, but similar multi-band effects may contribute to the observed layer dependence of kinetic inductance in NbSe_2_ (Fig. 4c). Additionally, as the superconducting gap (or *T*_c_) decreases with reduced thickness, the superconducting coherence length becomes longer. For a fixed mean free path, this leads to a larger coherence length to mean free path ratio, indicating a shift toward dirty-limit behavior in the thinner samples. We note that the combined contributions from both clean- and dirty-limit superconductivity observed in the microwave measurements here contrast with previous DC transport studies, which reported only clean-limit behavior. This discrepancy suggests that the microwave response may be sensitive to the multi-band nature of NbSe_2_^41,49–51^.

Finally, in thin superconducting films where the thickness *d* is much smaller than the penetration depth *λ*, the relevant length scale becomes the Pearl length $\Lambda=\frac{2{\lambda }^{2}}{d}$^70^. All of our NbSe_2_ samples operate in this Pearl regime (*d* ≪ *λ* ~ 230 nm^71^), with both thickness and width much smaller than the estimated Pearl length (10–70 μm; see Supplementary Note 5). Recent studies on high-*T*_c_ YBCO superconductors have shown that geometric confinement in this regime can strongly enhance the kinetic inductance as the device width approaches or falls below *Λ*^72^. While a detailed investigation of this effect in NbSe_2_ lies beyond the scope of this work, our observation of large *L*_k_ values—and a steeper-than-expected inverse thickness scaling, relative to the prediction of Eq. (2)—may be qualitatively consistent with such confinement-enhanced kinetic inductance in the Pearl regime.

We characterize the kinetic inductance and nonlinear properties of NbSe_2_ using a coplanar waveguide resonator terminated by hBN-encapsulated NbSe_2_ thin films. The kinetic inductance shows an approximate 1/*d*-type scaling for the thinner samples and reaches 1.2nH/□ at monolayer thickness—among the highest reported for superconductors within this thickness regime. Under the microwave drive, the device exhibits a nonlinear frequency shift that is consistent with the nonlinear Kerr model. The extracted Kerr coefficients (*K*/2*π* = −0.006 to 14.7 Hz/photon) are relatively small, making NbSe_2_ well-suited for applications that require large linear inductance with minimal nonlinearity, such as the linear shunt-inductor used in fluxonium qubits and in photon detectors operating in the microwave regime^73^. The *L*_k_ exhibits a quadratic dependence on DC bias current, consistent with the Ginzburg-Landau framework. The measured kinetic inductance in NbSe_2_ exhibits a crossover from clean- to dirty-limit behavior as the thickness decreases. This transition can be qualitatively described using a generalized expression for *L*_k_ derived from BCS theory. In the ultra-thin regime, enhanced contributions from the dirty limit may arise due to increased surface scattering, multi-band superconductivity, and geometric confinement. We demonstrate that NbSe_2_ is a crystalline two-dimensional superconductor that exhibits large, linear inductance. The hBN–NbSe_2_–hBN heterostructure is well suited for compact lump-element devices, such as shunt inductors for fluxonium qubits or readout resonators, while minimizing parasitic coupling. These results also highlight the ability of microwave measurements to probe superconducting properties in low-dimensional materials, offering a valuable complement to DC transport and other techniques.

## Methods

### Device assembly and fabrication

Aluminum CPW resonators are fabricated from a highly resistive 2-inch Si wafer coated with 250 nm MBE-grown aluminum film. The resonator circuit is patterned via photolithography and wet etched using Transene Aluminum etchant. Afterward, the 2-inch wafer is diced into 5 mm × 5 mm chips. The bulk crystal of NbSe_2_ is grown by HQ Graphene. The mechanical exfoliation and encapsulation of thin NbSe_2_ layers with hBN are carried out inside an Argon-filled glove box, maintaining oxygen and water levels below 0.1 ppm. This prevents oxidation of NbSe_2_, keeping the devices within the low-disorder regime. The hBN–NbSe_2_–hBN heterostructures are transferred on the chip using dry polymer-based techniques^74–76^. After transferring the hBN–NbSe_2_–hBN stacks onto the pre-patterned chips, the van der Waals heterostructures are taken out of the glove box. To process edge-contact, first, the stack goes through reactive-ion etching with O_2_/Ar/CHF_3_ to open edges of NbSe_2_. The samples are immediately loaded into an e-beam evaporator to do in-situ ion-milling to clean the exposed edges. Aluminum is then deposited onto the cleaned NbSe_2_ edge at a tilt angle of 30^∘^ with the substrate rotating at 60 rpm, ensuring uniform coverage^55^. The bridging between the contact aluminum and the pre-fabricated aluminum resonator and ground plane is done by another in-situ ion mill and a normal-incidence aluminum deposition^77^.

### Measurement setup

The experiment was performed using a Bluefors XLD1000 dilution refrigerator with a base electron temperature of 10 mK. To send the microwave signals down to the package, we use semi-rigid coaxial cables with low thermal conductivity and low-loss superconducting niobium–titanium (NbTi) cables for the readout. Within our readout input lines, we have integrated attenuation mechanisms: a 20 dB attenuator at 4 K, a 10 dB attenuator at the still plate, and a 40 dB attenuator at the mixing chamber stage. The output signals pass through a 2 GHz high-pass filter and a 12 GHz low-pass filter. The readout chain incorporates a Josephson traveling-wave parametric amplifier (TWPA), whose pump tone is combined with the readout signal using a directional coupler. The output signal is subsequently amplified by a low-noise high-electron-mobility transistor (HEMT) amplifier at the 4 K stage, followed by room-temperature amplification. DC lines connected to the device are filtered at 4 K with an RC *π*-filter and an RF + RC filter with a cut-off frequency of 50 kHz at the mixing chamber stage.

DC transport measurements were performed using standard low-frequency lock-in techniques. A typical AC excitation current of 1 nA at 17.77 Hz was applied to the sample, while the resulting AC voltage drop was measured simultaneously. A DC bias current was applied using a multichannel low-noise voltage source (QDAC from Qdevil). Microwave transmission measurements were performed using a Keysight N5232A VNA to send and receive the microwave signal. The control electronics used in this experiment are listed in Supplementary Table 2.

### Extraction of kinetic inductance from resonance frequency

To extract the kinetic inductance (*L*_k_) of NbSe_2_, we employ a comparative model using *λ*/4-resonators terminated with aluminum and NbSe_2_. For the aluminum-terminated resonator with a length of *l* and a resonant frequency of *f*_r,Al_, the input impedance *Z*_Al_ is expressed as^78^

3 $${Z}_{{{{\rm{Al,in}}}}}={Z}_{0}\tanh (\alpha+i\beta )l$$

where *Z*_0_ is the characteristic impedance which is 50 Ω in our work, *α* and *β* are the attenuation constant and phase constant respectively. For a lossless line, the attenuation constant *α* is zero, and *β*(*f*) becomes

4 $$\beta ( \; f)=\frac{2\pi {f}\sqrt{{\epsilon }_{{\rm {eff}}}}}{c}$$

For the *λ*/4-resonator terminated by NbSe_2_, the kinetic inductance (*L*_k_) contributes an additional frequency-dependent term in the impedance, which is modeled as

5 $${Z}_{{{{{\rm{Al-NbSe}}}}}_{{{{\rm{2,in}}}}}}={Z}_{0}\frac{i{\omega }_{{{{{\rm{r,Al-NbSe}}}}}_{{{{\rm{2}}}}}}{L}_{{{{\rm{k}}}}}+{Z}_{0}\tanh \left(i\beta l\right)}{{Z}_{0}+i{\omega }_{{{{{\rm{r,Al-NbSe}}}}}_{{{{\rm{2}}}}}}{L}_{{{{\rm{k}}}}}\tanh \left(i\beta l\right)}$$

The physical length *l* of the resonator is determined from the aluminum reference resonator using Eq. (3), assuming identical geometry and effective permittivity. On resonance, the impedance of a *λ*/4-resonator diverges; for the NbSe_2_-terminated case, Eq. (5) reduces to

6 $$\tan \left(\frac{{\omega }_{{{{{\rm{r,Al-NbSe}}}}}_{{{{\rm{2}}}}}}\sqrt{{\epsilon }_{{\rm {eff}}}}}{c}l\right)=\frac{{Z}_{0}}{{\omega }_{{{{{\rm{r,Al-NbSe}}}}}_{{{{\rm{2}}}}}}{L}_{{{{\rm{k}}}}}}$$

We extract the kinetic inductance *L*_k_ of NbSe_2_ from Eq. (6), where the resonant frequency of NbSe_2_-terminated resonator is in the linear regime (in the single-photon limit).

## Supplementary information

Supplementary Information

Transparent Peer Review file

## Data Availability

The data that support the findings of this study are available from the corresponding author upon request and with the cognizance of our US Government sponsors, who funded the work.
