# Supplementary material for: Kinetic inductance of few-layer NbSe2 in the two-dimensional limit
Source: Nat Commun. 2026 Jul 23;17:9007. doi: 10.1038/s41467-026-75672-8 (PMC13507152; doi:10.1038/s41467-026-75672-8)
Supplement: Supplementary file 1 [file 41467_2026_75672_MOESM1_ESM.pdf]

Supplementary Information

Kinetic Inductance of Few-Layer $NbSe_2$ in the Two-Dimensional Limit

Sameia Zaman[1,2*], Joel Î-j. Wang[1,3*], Thomas Werkmeister[4], Miuko Tanaka[1], Thao Dinh[5], Max Hays[1], Daniel Rodan-Legrain[1], Aranya Goswami[1], Réouven Assouly[1], Ahmet Kemal Demir[5], David K. Kim[6], Bethany M. Niedzielski[6], Kyle Serniak[1,6], Mollie E. Schwartz[6], Kenji Watanabe[7], Takashi Taniguchi[8], Philip Kim[4,9], Riccardo Comin[5], Jeffrey A. Grover[1], Terry P. Orlando[1,2], Pablo Jarillo-Herrero[5], and William D. Oliver[1,2,5*]

[1] *Research Laboratory of Electronics, Massachusetts Institute of Technology, Cambridge, MA 02139, USA*

[2] *Department of Electrical Engineering and Computer Science, Massachusetts Institute of Technology, Cambridge, MA 02139, USA*

[3] *Department of Physics, New York University, NY 10003, USA*

[4] *John A. Paulson School of Engineering and Applied Sciences, Harvard University, Cambridge, MA, USA*

[5] *Department of Physics, Massachusetts Institute of Technology, Cambridge, MA 02139, USA*

[6] *Lincoln Laboratory, Massachusetts Institute of Technology, Lexington, MA 02421, USA*

[7] *Research Center for Electronic and Optical Materials, National Institute for Materials Science, 1-1 Namiki, Tsukuba 305-0044, Japan*

[8] *Research Center for Materials Nanoarchitectonics, National Institute for Materials Science, 1-1 Namiki, Tsukuba 305-0044, Japan*

[9] *Department of Physics, Harvard University, Cambridge, MA, USA*

*To whom correspondence should be addressed: zsameia@mit.edu, joelwang@mit.edu, and william.oliver@mit.edu

Supplementary Note 1:

Contact Resistance between hBN-encapsulated $NbSe_2$ and Al

It is critical to establish a low-resistance, transparent contact (with near-zero contact resistance) between hBN encapsulated $NbSe_2$ and the aluminum electrode for achieving impedance matching and accurate resonant frequency measurements. A low contact resistance (R_c) is required because if it exceeds the characteristic impedance ($50\,\Omega$ for our case), the resonator behaves like a $\lambda/2$-resonator instead of the desired $\lambda/4$ mode terminated with $NbSe_2$ sample. This happens because the higher resistance causes the end of the resonator to act like an open circuit, disrupting the intended resonant behavior. Additionally, minimizing R_c reduces dissipation and enables high-Q, high-accuracy measurements for characterizing the kinetic inductance and other superconducting properties of $NbSe_2$.

We fabricate a few devices for DC measurement using the above fabrication procedures to confirm a reliable superconducting contact between thin $NbSe_2$ and Al. We vary the ion-milling duration to determine the optimal time needed to remove oxides from the edges of $NbSe_2$. To eliminate the line resistances, we measure the devices in a pseudo-4-probe configuration, Supplementary Figure 1a. Below the transitions of both $NbSe_2$ and Al, the total resistance of the device, including the Al-$NbSe_2$ junctions, drops to zero within the noise floor of our measurement ($R_\mathrm{c} = 0.01\,\Omega$) Supplementary Figure 1b-c. We present DC transport measurements to characterize the contact resistance as a function of ion milling time, with the results summarized in Supplementary Table 1. We selected 105 s for the ion milling time, which we demonstrated to provide a reproducible and low contact resistance.

**Ion-milling time (sec)**	$\boldsymbol{R}_\mathrm{c}$ **(Ω/μm)**	$\boldsymbol{R}_\mathrm{s}$ **(Ω/sq)**	$\boldsymbol{T}_\mathrm{c}$ **(K)**	$\boldsymbol{d}$ **(nm)**
120	0.01	N/A	N/A	10
105	0.01	31	5.5	6
90	0.01	19	7	8
90	0.02	N/A	N/A	4
80	1,80	60	4.8	4
75	0.02,2	29	5.5	6
60	0.02	45	4.9	5

Supplementary Table 1: **Summary of DC samples for contact resistance characterization.** The in-situ ion-milling time and the thickness of $NbSe_2$ both have been varied to characterize the contact resistance between $NbSe_2$ edge and aluminum interface.

The bulk $NbSe_2$ undergoes a transition into the charge-order phase at T $\sim$ 33 K, and a transition into the superconducting phase at T $\sim$ 7 K [1–3]. As the thickness of $NbSe_2$ decreases, the critical temperature (T_c) decreases, as shown in Supplementary Figure 1d, our results are consistent with other studies in the literature [4]. All our $NbSe_2$ samples are high-quality crystals with relatively high residual resistance ratios ($R_{300\mathrm{K}}/R_\mathrm{N}$

= 5-10) [1], the normal-state sheet resistance (R_s) right above the superconducting transition increases as the thickness of $NbSe_2$ decreases; the values are summarized in Supplementary Table 1. Supplementary Figure 1e-f shows four-terminal transport characterization of one of the devices ($d = 8\,\mathrm{nm}$) using current biasing. The differential resistance was measured by applying a small AC excitation current (1 nA) superimposed on a DC bias current and detecting the AC voltage (dV) between two electrodes. The critical current of the $NbSe_2$ sample is of order 0.4 mA, which is consistent with the previous study [5, 6].

Component	Manufacturer	Model
Dilution Refrigerator	Bluefors	XLD1000
VNA	Keysight	N5232A
DC Source	QDevil	QDAC
Lock-in Amplifier	Stanford Research Systems	SR830
Multimeter	Keithley	2010

Supplementary Table 2: **Summary of control electronics.** Specifications of the control equipment used in the experiment, with their manufacturer and Model numbers.

Supplementary Note 2: Resonator Circular Fit

For notch-type geometry, where the resonator is coupled to a transmission line, the frequency-dependent transmission S_{21} is given by [7, 8]:

$$S_{21}^{notch}(f) = ae^{i\alpha}e^{-2\pi i f\tau}\left[1 - \frac{\left(\frac{Q_\mathrm{L}}{|Q_\mathrm{c}|}\right)e^{i\phi}}{1 + 2iQ_\mathrm{L}\left(\frac{f}{f_\mathrm{r}} - 1\right)}\right] \tag{1}$$

where f is the signal frequency, f_r is the resonance frequency, Q_L is the loaded quality factor, Q_c is the coupling quality factor, and ϕ corresponds to the phase shift of resonance due to the impedance mismatch with the transmission line. The prefactor ($ae^{i\alpha}$) includes the overall gain and phase delay of the transmission line, including all the attenuation and amplification. Additionally, τ denotes the propagation time in the transmission line, which introduces a phase shift proportional to the frequency f. Supplementary Figure 3 shows the raw data (blue) and the Lorentzian fit using Eq. 1 (red) of the spectator resonator in the single-photon limit. We extract the values of the loaded quality factor (Q_L) and the coupling quality factor (Q_c) from the fit. The internal loss determines the intrinsic quality (Q_i) of the resonator extracted from: $\frac{1}{Q_\mathrm{L}} = \frac{1}{Q_\mathrm{i}} + \frac{1}{Q_\mathrm{c}}$. The fitted internal quality factors (Q_i) of six different $\lambda/4$-resonators are plotted in Supplementary Figure 4c.

Supplementary Note 3: Non-linearity of the Resonator from the Inductance

We observe the resonant frequency shift in the $NbSe_2$-terminated resonator at higher microwave power (Fig. 3, main text), while the Al-terminated resonator does not show this behavior (Supplementary Figure 4b). The resonant frequency shift with increasing photon number in the resonator is described by the Kerr Hamiltonian [9–11]

$$H = \hbar\omega_{\mathrm{r}} a^{\dagger} a + \frac{\hbar}{2} K a^{\dagger} a^{\dagger} a a \tag{2}$$

where $\hbar$ is the reduced Planck constant, $a^{\dagger}$ and a are the creation and annihilation operators, ω_{r} is the resonant frequency, and K is the Kerr constant, which is a measure of the strength of the nonlinearity in the system. The Kerr nonlinearity shifts the resonance frequency and is proportional to the resonator photon number. In addition to the power-dependent frequency shift, we observe a broadening of the internal linewidth as the number of photons in the cavity increases. The Kerr coefficient of $NbSe_2$ is determined by analyzing the relationship between the resonance frequency shift (Δf) and the resonator photon number (n_{r}) in the resonator. We calculate the photon number in the resonator as a function of the applied power using the following formula [12],

$$n_{\mathrm{r}} = \frac{2\kappa P_{\mathrm{rf}}}{\hbar\omega_{\mathrm{r}}(\kappa+\gamma)^2} \tag{3}$$

Here, κ represents the coupling rate to the feedline, P_{rf} is the power applied at the resonator input, and γ is the internal loss rate.

Expanding the current-dependent kinetic inductance relation of Eq. 1 (main text), the quartic energy contribution is expressed as

$$U_{\mathrm{m}} = \frac{1}{2} L_{\mathrm{k}} \frac{I_{\mathrm{DC}}^4}{I^{*2}} \tag{4}$$

Here, I^{*} sets the scale for the non-linearity of the film. Applying circuit quantization, the current operator can be expanded in terms of ladder operators as $\hat{I_{\mathrm{DC}}} = I_{\mathrm{zpf}}\,(a + a^{*})$ where $I_{\mathrm{zpf}} = \sqrt{\frac{\hbar\omega_{\mathrm{r}}}{2L_0}}$ is the zero-point current fluctuation of the mode and L_0 is the total inductance of the resonator mode. We obtain the

quantized nonlinear energy term of Eq. 4 as

$$\hat{U}m = \frac{1}{2} L_k \frac{Izpf^4}{I^{*2}} (a + a^*)^4 \tag{5}$$

The Kerr nonlinearity arises from the term proportional to a^*a^*aa in the quartic expansion, and the resulting Kerr coefficient (K) is

$$K = \frac{6L_\mathrm{k} I_\mathrm{zpf}^4}{\hbar I^{*2}} = \frac{3\hbar\omega_\mathrm{r}^2}{2L_0^2} \cdot \frac{L_\mathrm{k}}{I^{*2}} \tag{6}$$

For device D6 (shown in Fig. 3 of the main text), we calculate the Kerr coefficient to be $K/2\pi = -3.8$ Hz/photon using Eq. 6, which is in good agreement with the value ($K/2\pi = -1.7$) extracted from the power-dependent frequency shift. The calculation uses $f_{\mathrm{r,Al-NbSe_2}} = 4.79\,\mathrm{GHz}$, $L_0 = L_\mathrm{g} + L_\mathrm{k} = 2.627\,\mathrm{nH}$, and $I^* = 0.4\,\mathrm{mA}$.

Supplementary Note 4: Kinetic Inductance in Clean and Dirty Limits

The purity of a superconductor is characterized by the ratio of the mean free path (l_MFP) of the Cooper pairs to the BCS coherence length (ξ_0). The mean free path (l_MFP) is defined as $l_\mathrm{MFP} = \tau v_\mathrm{F}$ with τ representing the time interval between collisions in the normal state of the superconductor and v_F denoting the Fermi velocity [13]. The superconductor is in the dirty metal limit if the mean free path (l_MFP) is much shorter than the BCS coherence length (ξ_0), and in the clean limit if l_MFP is much longer than ξ_0 [14]. To obtain kinetic inductance for both the dirty and clean limit, two pieces of information are required: (1) the generalized first London equation for a superconductor relating current density and electric field, and (2) an expression characterizing the penetration depth (specifically in the dirty/clean limit). The first London equation establishes a direct relation between the supercurrent density $\mathbf{J}$ and the electric field $\mathbf{E}$,

$$\frac{\partial \mathbf{J}}{\partial t} = \frac{1}{\mu_0 \lambda^2} \mathbf{E} \tag{7}$$

where λ is the penetration depth and μ_0 is the permeability of free space. When combined with Maxwell's equations, the London formalism further predicts that magnetic fields decay exponentially inside a super-

conductor, with the characteristic length λ. With a sinusoidal drive of frequency ω, Eq. 7 gives

$$i\omega\left(\mu_0\lambda^2\right)J = E \tag{8a}$$

$$i\omega\left(\mu_0\lambda^2\frac{l}{A}\right)I = V \tag{8b}$$

Equation 8b follows from the substitutions $V = El$ and $I = JA$, and the prefactor in the parentheses represents the kinetic inductance.

$$L_{\mathrm{k}} = \mu_0\lambda^2\frac{l}{A} \tag{9}$$

Equation 9 depends on the phenomenological parameter λ, which needs to be evaluated in both clean and dirty limits.

Supplementary Note 4.1: Clean Limit

In the clean limit, where the mean free path of the superconducting carriers is much longer than the coherence length, scattering events can be neglected. In this case, the dynamics of the supercurrent are determined solely by the acceleration of Cooper pairs under the applied electric field. This leads to the relation

$$\frac{d}{dt}(m_{\mathrm{eff}}\mathbf{v}) = \frac{d}{dt}\left(\frac{m_{\mathrm{eff}}}{n_0e^2}\mathbf{J}\right) = \mathbf{E} \tag{10}$$

Here, n_0 is the carrier density and m_{eff} is the effective mass of the supercurrent carriers. Equation 10 allows us to identify the penetration depth (λ_{clean}) and kinetic inductance ($L_{\mathrm{k,clean}}$) in the clean limit as,

$$\mu_0\lambda_{clean}^2 = \frac{m_{\mathrm{eff}}}{n_0e^2} \tag{11}$$

$$L_{\mathrm{k,clean}} = \frac{m_{\mathrm{eff}}}{n_0e^2}\left(\frac{l}{A}\right) \tag{12}$$

This penetration depth is known as the London penetration depth ($\lambda_{\mathrm{clean}} = \lambda_{\mathrm{L}}$).

Supplementary Note 4.2: Dirty Limit

In the dirty limit, impurity scattering shortens the mean free path of the carriers, which weakens superfluid screening and causes the magnetic field to penetrate deeper, leading to an enhanced penetration depth given by [13, 15],

$$\lambda_{\mathrm{dirty}} \approx \lambda_{\mathrm{L}}\left(\frac{\xi_0}{l_{\mathrm{MFP}}}\right)^{1/2} \tag{13}$$

where ξ_0 is the BCS coherence length at zero temperature $\left(\xi_0 = \frac{\hbar v_{\mathrm{F}}}{\pi\Delta_0} = \xi_{\mathrm{clean}}\right)$ and the coherence length in the dirty limit becomes,

$$\xi_{\mathrm{dirty}} \approx \xi_0 \left(\frac{l_{\mathrm{MFP}}}{\xi_0}\right)^{1/2} = (\xi_0 l_{\mathrm{MFP}})^{1/2} \tag{14}$$

Plugging the expression of λ_{dirty} into the general expression for L_{k} in Eq. 9, we get

$$L_{\mathrm{k,dirty}} = \mu_0 \lambda_{\mathrm{dirty}}^2 \frac{l}{A} = \mu_0 \lambda_{\mathrm{L}}^2 \left(\frac{\xi_0}{l_{\mathrm{MFP}}}\right) \frac{l}{A} \tag{15}$$

Now, using the expression of the BCS coherence length ($\xi_0 = \hbar v_{\mathrm{F}}/\pi\Delta_0$) and London penetration depth (λ_{L}), we obtain

$$L_{\mathrm{k,dirty}} = \frac{m_{\mathrm{eff}}}{n_0 e^2} \frac{\hbar v_{\mathrm{F}}}{\pi\Delta_0} \frac{l}{l_{\mathrm{MFP}} A} \tag{16}$$

Equation 16 can be further simplified by substituting the expressions for mean free path ($l_{\mathrm{MFP}} = v_{\mathrm{F}}\tau$) and resistivity ($\rho_{\mathrm{s}} = 1/\sigma$).

$$L_{\mathrm{k,dirty}} = \rho_{\mathrm{s}} \frac{\hbar}{\pi\Delta_0} \frac{l}{A} = R_{\mathrm{s}} \frac{\hbar}{\pi\Delta_0} \frac{l}{w} \tag{17}$$

where R_{s} is the normal-state sheet resistance of a current-carrying superconducting wire of length l, width w, and film thickness d. The BCS superconducting gap is approximated to $\Delta_0 \approx 1.764 k_{\mathrm{B}} T_{\mathrm{c}}$ for $T \ll T_{\mathrm{c}}$ with T_{c} the critical temperature and Eq. 17 becomes

$$L_{\mathrm{k,sq,dirty}} = \frac{\hbar}{1.764\pi k_{\mathrm{B}}} \frac{R_{\mathrm{s}}}{T_{\mathrm{c}}} \tag{18}$$

Layers	$L_{\mathrm{k,sq}}$ (pH/sq)	T_{c} (K)	R_s (Ω/sq)	$R_{\mathrm{s}}/T_{\mathrm{c}}$ (Ω/K)	$L_{k,sq}/L_{k,sq,clean}$
1	1195	3.5 [4]	240 [4]	68.57	14.95
4	282	4.8	60	12.5	3.53
6	191	5.5	29	5.27	2.39
7	143	6.0	25	4.17	1.79
8	135	7	19	2.71	1.79
8	135	6.3 [3]	14 [3]	2.22	1.75
10	56	7.0 [4]	10 [4]	1.43	0.71

Supplementary Table 3: **Kinetic inductance of $NbSe_2$ from clean to dirty limit**

Supplementary Note 4.3: Full Range Calculation from Clean to Dirty Limit

We can calculate the kinetic inductance for arbitrary ξ_0/l_{MFP} (where the ratio of ξ_0/l_{MFP} is of the order of 1). The Ginzburg-Landau theory gives a penetration depth of the form which can be well approximated

by (Eq. 3.123b in [13] and the appendix in [16]):

$$\lambda = \lambda_{\mathrm{L}} \left(1 + a\frac{\xi_0}{l_{MFP}}\right)^{1/2} \tag{19a}$$

$$L_{\mathrm{k}} = \mu_0 \lambda_{\mathrm{L}}^2 \left(1 + a\frac{\xi_0}{l_{MFP}}\right)\left(\frac{l}{A}\right) \tag{19b}$$

$$L_{k,sq} = \mu_0 \frac{\lambda_{\mathrm{L}}^2}{d} + \mu_0 \frac{\lambda_{\mathrm{L}}^2}{d}\left(a\frac{\xi_0}{l_{MFP}}\right) \tag{19c}$$

The factor a in Eq. 19a-c is the order of unity ($a = 0.75$), but we have set a to be unity in the main text for simplicity. Equation 19c indicates that the kinetic inductance per square is the sum of the clean and dirty limit results. We can extract the ratio of coherence length to mean free path ($\frac{\xi_0}{l_{MFP}}$) from the above expression.

$$\frac{\xi_0}{l_{MFP}} = \frac{L_{k,sq}}{L_{k,sq,clean}} - 1 \tag{20}$$

Supplementary Figure 5 shows the clean- and dirty-limit contributions to the measured sheet kinetic inductance $L_{\mathrm{k,sq}}$ of $NbSe_2$ samples, highlighting the $1/d$ dependence of the clean-limit term in thicker samples and the dominant dirty-limit contributions in thinner samples.

Supplementary Figure 5 plots results from the third expression in Eq. 2 (main text) which is of the form $L_{\mathrm{k,sq}} = \alpha/d + \beta\frac{R_{\mathrm{s}}}{T_{\mathrm{c}}}$. Supplementary Figure 5a plots $L_{\mathrm{k,sq}} \times d$ versus ρ/T_{c} where $\rho = R_{\mathrm{s}}d$. The y-intercept of Supplementary Figure 5a is used to get an estimate of α. Supplementary Figure 5b uses this value of α to plot, $L_{\mathrm{k,sq}} - \alpha/d$ versus $R_{\mathrm{s}}/T_{\mathrm{c}}$, where the y axis now shows the contribution to $L_{\mathrm{k,sq}}$ from the dirty limit. Given that α/d is about 70 pH for the 10-layer sample, the values for the thicker samples have smaller contributions from the dirty-limit term and are thus mostly in the clean limit. The samples for high sheet resistance are dominated by the dirty-limit contribution.

Supplementary Note 5: Pearl Length

In superconducting films where the thickness d is much smaller than the penetration depth λ_L ($d \ll \lambda$), magnetic fields are weakly screened in the transverse direction and instead spread laterally over long distances. This lateral field penetration is characterized by the Pearl length, defined as

$$\Lambda = \frac{2\lambda^2}{d} \tag{21}$$

The Pearl lengths of all the devices in this work are estimated using Eq. 21, where the penetration depth of $NbSe_2$ is 230 nm.

Supplementary Note 6: Determination of $NbSe_2$ Thickness and Layer Number

Supplementary Note 6.1: AFM Characterization of hBN-$NbSe_2$-hBN Stack

Atomic force microscopy (AFM) is a high-resolution imaging technique used in this work to characterize the surface topography and height of hBN-$NbSe_2$-hBN stacks. The thickness of hBN encapsulated $NbSe_2$ is determined by tapping-mode AFM, illustrated in Supplementary Figure 8. The AFM measurement tends to overestimate the actual thickness of $NbSe_2$ due to the presence of the top hBN encapsulation. In this work, the effective $NbSe_2$ thickness is slightly smaller, as confirmed by the shear mode in the low-frequency Raman spectrum.

Supplementary Note 6.2: Raman Spectroscopy of hBN-$NbSe_2$-hBN Stack

The number of layers in our samples was determined by the shear mode frequency of Raman spectroscopy [17, 18]. The Raman data were collected using a Horiba LabRAM Evolution system in the XY channel at room temperature. A 50x objective (Mitutoyo MY50X-825) was employed to direct and collect light in the backscattering geometry with a 532 nm wavelength. Data were captured using a liquid nitrogen-cooled CCD, maintained at -133°C. The incident laser power on the sample was estimated to be below 4 mW. Supplementary Figure 7b shows that the low-frequency mode observed in the Raman spectra corresponds to interlayer shear modes. In all spectra, shear modes are analyzed using Lorentzian peak fitting after subtracting the background. The shear mode peak in Raman spectra originates from interlayer shearing and is absent in the monolayer sample. The frequency of the shear modes (ω_s) decreases with decreasing layer number (N), reflecting a reduced effective interlayer spring constant. The relationship can be described as $\omega_\mathrm{s} = \omega_\mathrm{s,Bulk}\cos\left(\frac{\pi}{2N}\right)$ with a bulk shear mode frequency $\omega_\mathrm{s,Bulk} = 29.39\,\mathrm{cm}^{-1}$ [18, 19]. The AFM-measured thicknesses of the $NbSe_2$ flakes are consistent with the layer numbers determined from the shear mode frequency (1 layer = 1 nm) for all samples except for the thinnest device D1 (see main text Table 1). For D1, the AFM measurement indicates a thickness of 2 nm, while Raman spectroscopy only shows a weak feature at the frequency $19.04\,\mathrm{cm}^{-1}$ rather than a well-defined Lorentzian peak (Supplementary Figure 7b). This corresponds to a layer number less than 2, with a fitted value of N =1.5 (Supplementary Figure 7a). We use this Raman-derived value (1.5 nm) in the analysis presented in Fig. 4a (main text).

Supplementary References

1. Hamill, A. *et al.* Two-fold symmetric superconductivity in few-layer $NbSe_2$. *Nature physics* **17,** 949–954 (2021).
2. Sanna, A. *et al.* Real-space anisotropy of the superconducting gap in the charge-density wave material 2H-$NbSe_2$. *npj Quantum Materials* **7,** 6 (2022).
3. Cao, Y. *et al.* Quality heterostructures from two-dimensional crystals unstable in air by their assembly in inert atmosphere. *Nano Letters* **15,** 4914–4921 (2015).
4. Khestanova, E. *et al.* Unusual suppression of the superconducting energy gap and critical temperature in atomically thin $NbSe_2$. *Nano Letters* **18,** 2623–2629 (2018).
5. Shein, K. *et al.* Fundamental limits of few-layer $NbSe_2$ microbolometers at terahertz frequencies. *Nano Letters* **24,** 2282–2288 (2024).
6. Yokoi, M. *et al.* Negative resistance state in superconducting $NbSe_2$ induced by surface acoustic waves. *Science Advances* **6,** eaba1377 (2020).
7. Probst, S., Song, F., Bushev, P. A., Ustinov, A. V. & Weides, M. Efficient and robust analysis of complex scattering data under noise in microwave resonators. *Review of Scientific Instruments* **86** (2015).
8. Baity, P. G. *et al.* Circle fit optimization for resonator quality factor measurements: Point redistribution for maximal accuracy. *Physical Review Research* **6,** 013329 (2024).
9. Reagor, M. J. *Superconducting cavities for circuit quantum electrodynamics* (Yale University, 2016).
10. Yurke, B., Buks, E., *et al.* Performance of cavity-parametric amplifiers, employing Kerr nonlinearites, in the presence of two-photon loss. *Journal of Lightwave Technology* **24,** 5054–5066 (2006).
11. Nayfeh, A. H., Mook, D. T., *et al. Nonlinear Oscillations* (John Wiley & Sons, 2008).
12. Norris, G. J. *et al.* Improved parameter targeting in 3D-integrated superconducting circuits through a polymer spacer process. *EPJ Quantum Technology* **11,** 5 (2024).
13. Tinkham, M. *Introduction to superconductivity* (Courier Corporation, 2004).
14. Parks, R. & Tinkham, M. *Superconductivity Vols. 1, 2* 1970.
15. Orlando, T. P. & Delin, K. A. *Foundations of Applied Superconductivity* (Addison-Wesley, 1991).
16. Orlando, T., McNiff Jr, E., Foner, S. & Beasley, M. Critical fields, Pauli paramagnetic limiting, and material parameters of Nb_3Sn and V_3Si. *Physical Review B* **19,** 4545 (1979).
17. Xi, X. *et al.* Strongly enhanced charge-density-wave order in monolayer $NbSe_2$. *Nature Nanotechnology* **10,** 765–769 (2015).
18. He, R. *et al.* Interlayer breathing and shear modes in $NbSe_2$ atomic layers. *2D Materials* **3,** 031008 (2016).

19. Lui, C. H., Ye, Z., Keiser, C., Xiao, X., He, R., *et al.* Temperature-activated layer-breathing vibrations in few-layer graphene. *Nano Letters* **14,** 4615–4621 (2014).

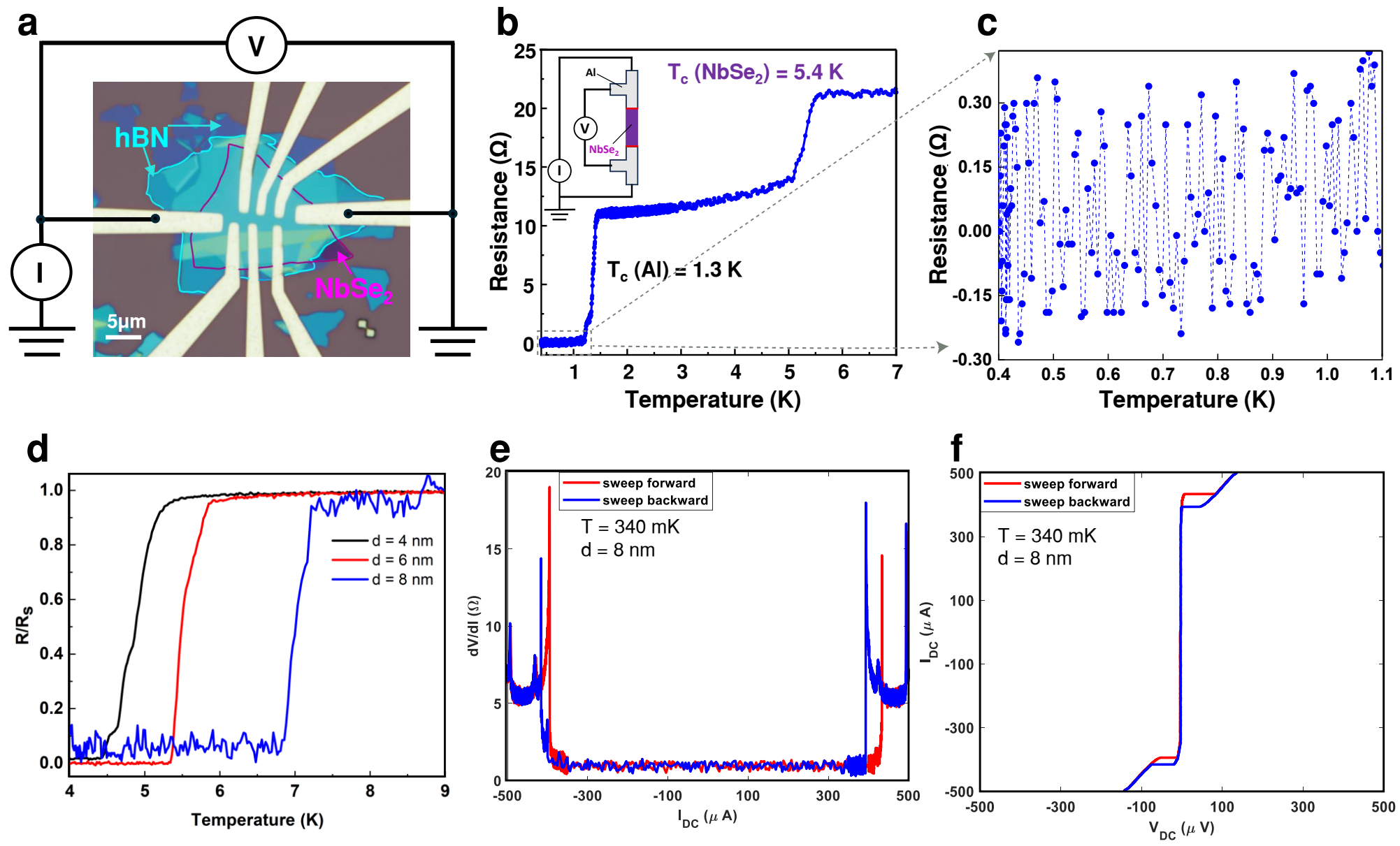

Supplementary Figure 1: **Al and $NbSe_2$ interface characterization and DC characteristics of $NbSe_2$.** **a,** Optical micrograph of a pseudo-4-probe DC device where hBN flakes fully encapsulate $NbSe_2$ flake, the current is applied between two terminals, and voltage is measured across those two terminals without taking into account the line resistance of the measurement setup. **b,** Resistance(T) for Al-$NbSe_2$-Al device showing the superconducting transition of $NbSe_2$ (d = 6 nm) at 5.4 K and the superconducting transition of Al at 1.3 K. Inset represents the 4-pt measurement setup for measuring the contact resistances of the Al/$NbSe_2$ interfaces. **c,** Total Resistance of the device below Al transition, which has dropped to zero within the noise floor of the measurement. **d,** Temperature dependence of the resistance for devices with $NbSe_2$ thicknesses of 4 nm, 6 nm, and 8 nm. To extract the T_c, we use the mean-field definition of T_c, which corresponds to half of the normal state resistance. **e,** Differential resistance (dV/dI) measurement for the d = 8 nm thick $NbSe_2$ of 4-point DC measurement. The plot shows the critical current 400 μA for that device. **f,** Current-voltage (I-V) relation on the same device.

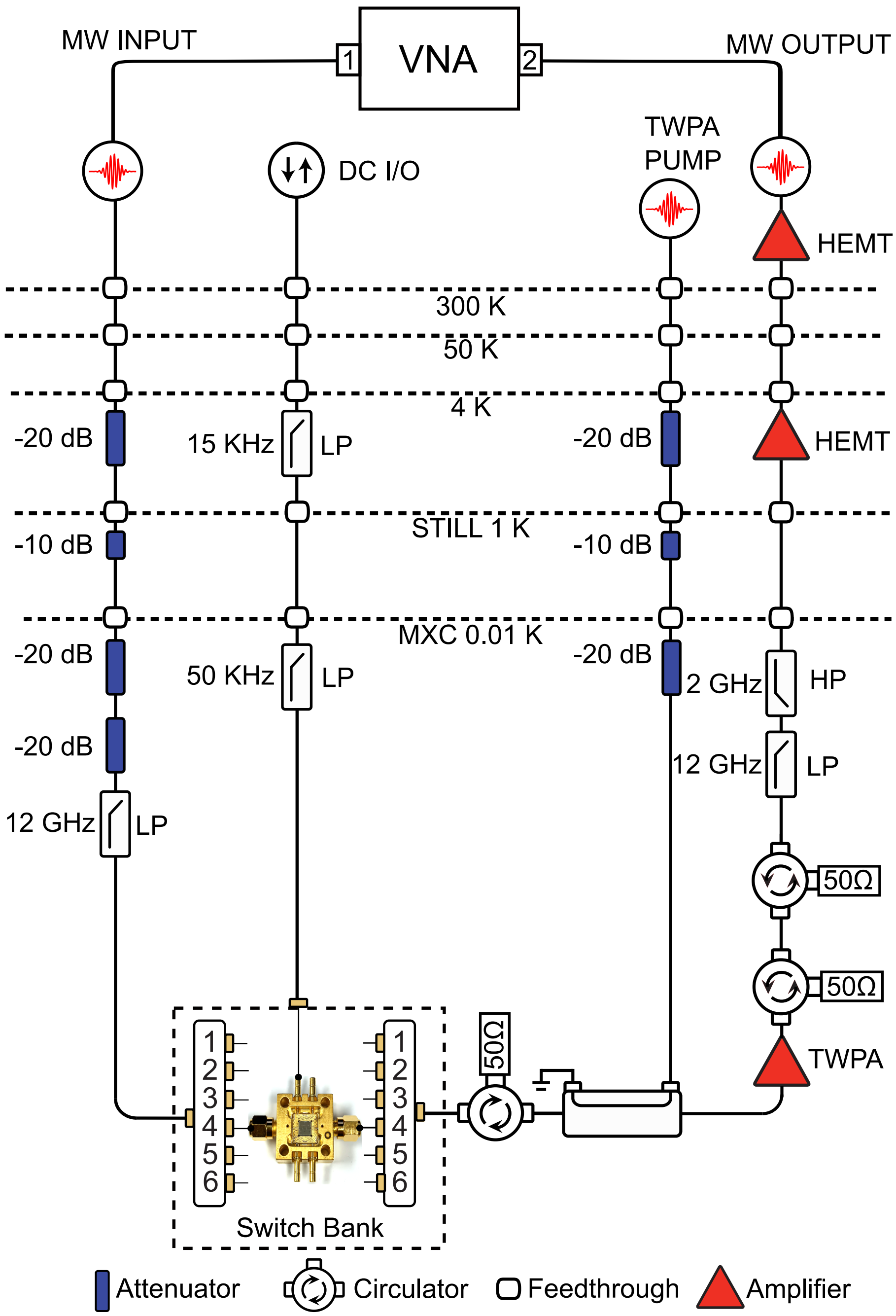

Supplementary Figure 2: **Measurement setup.** Wiring diagram of the microwave and DC setup to the device.

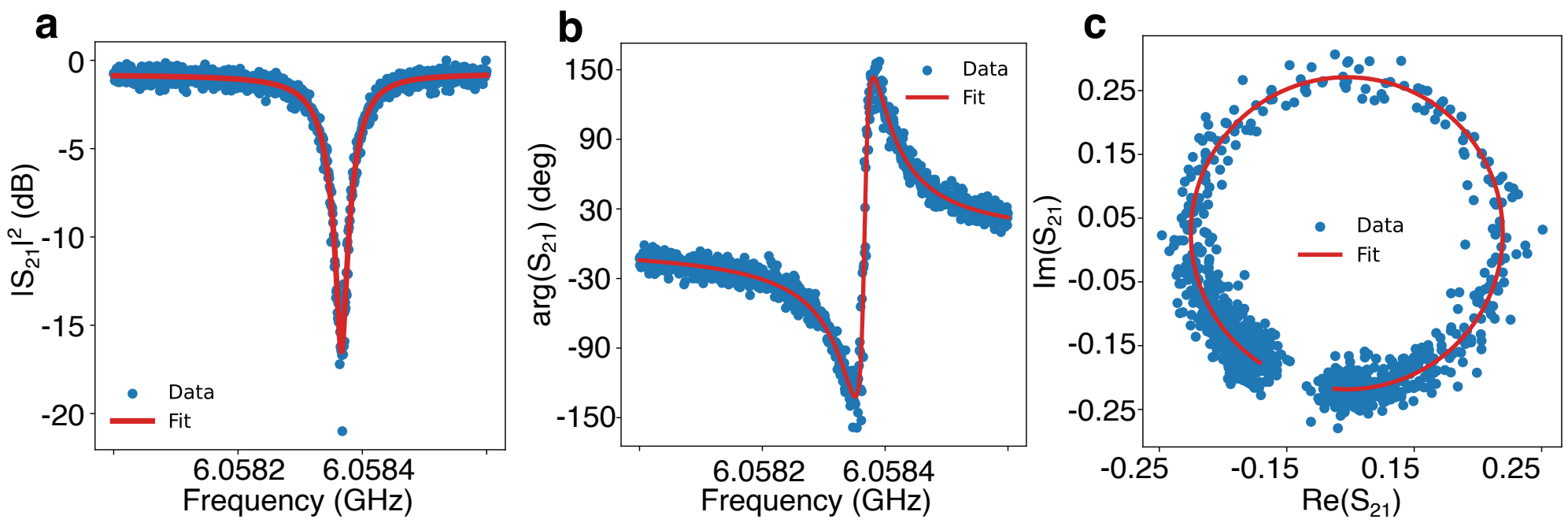

Supplementary Figure 3: **Raw data (blue) and Lorentzian fit (red) of Al "spectator" $\lambda/4$-resonator.a-b** Magnitude and phase response of S_{21} signal as a function of frequency at the single photon limit. The equation 1 function is used to fit the complex response. **c,** The imaginary part of S_{21} is plotted versus its real part, where the circle diameter corresponds to $Q_\mathrm{L}/Q_\mathrm{c} = \frac{Q_\mathrm{i}/Q_\mathrm{c}}{(Q_\mathrm{i}/Q_\mathrm{c})+1}$, which determines the contributions of coupling and internal losses of the resonator. The fitted resonance frequency is 6.0583 GHz, with an internal quality factor, Q_i is 5.985×10^6, and a coupling quality factor, Q_c is 10^5.

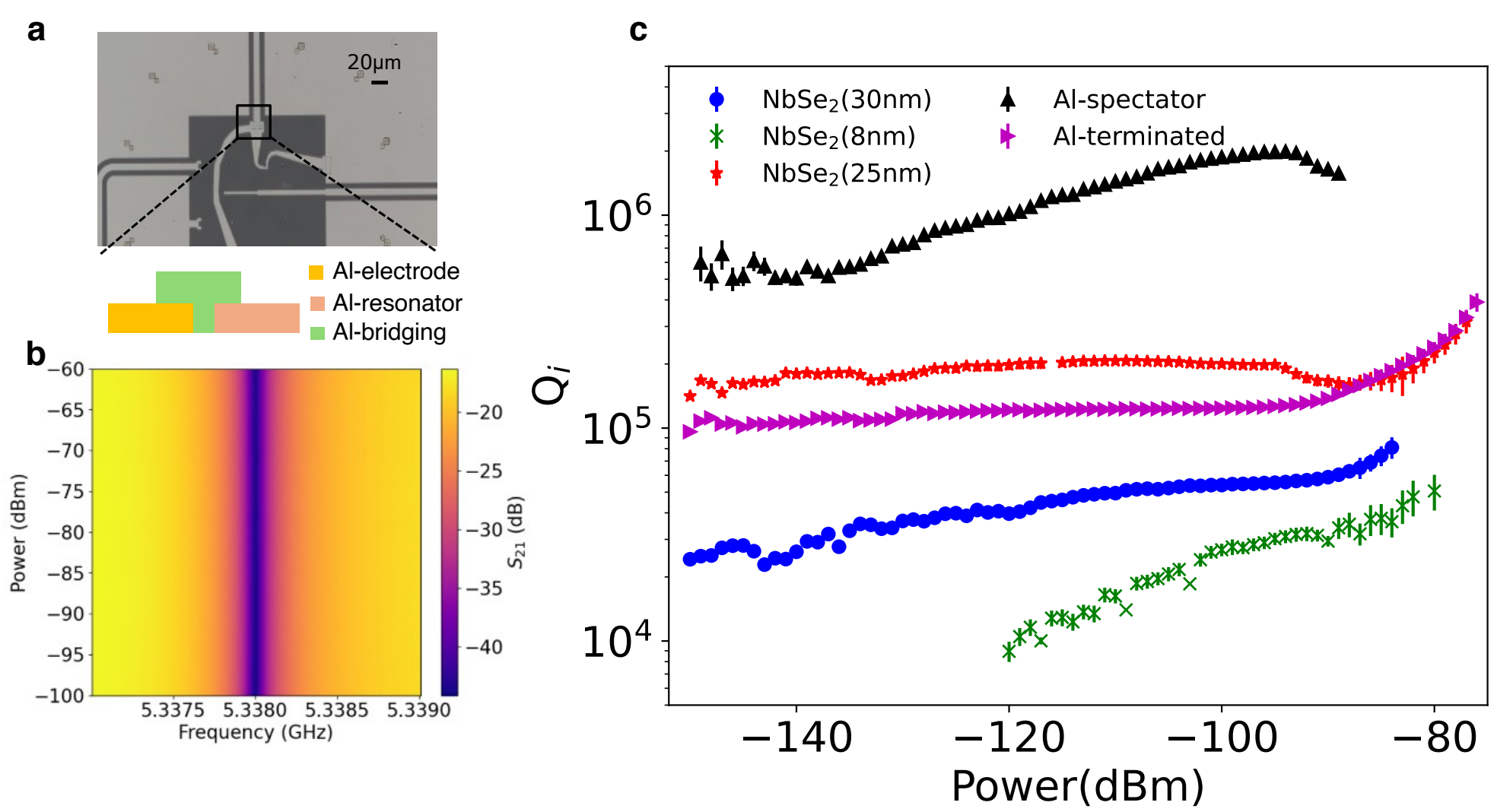

Supplementary Figure 4: **Measurements of $\lambda/4$ resonator. a,** Optical image of Al-terminated resonator of similar geometry used in Fig. 3 (main text). Here, two different Al layers (one MBE grown (orange) and another e-beam evaporated (yellow)) are made contact through an aluminum bridging step (green). The bridging step is performed by doing in-situ ion-milling to remove the native oxide layer and evaporate another layer of aluminum. **b,** Magnitude response of Al-shunted resonator with varying the input microwave power from -100 dBm to -60 dBm. The resonant frequency has a very negligible change, showing a small contribution of kinetic inductance from aluminum. **c,** Comparison of quality factors across different types of $\lambda/4$ resonators discussed in this work.

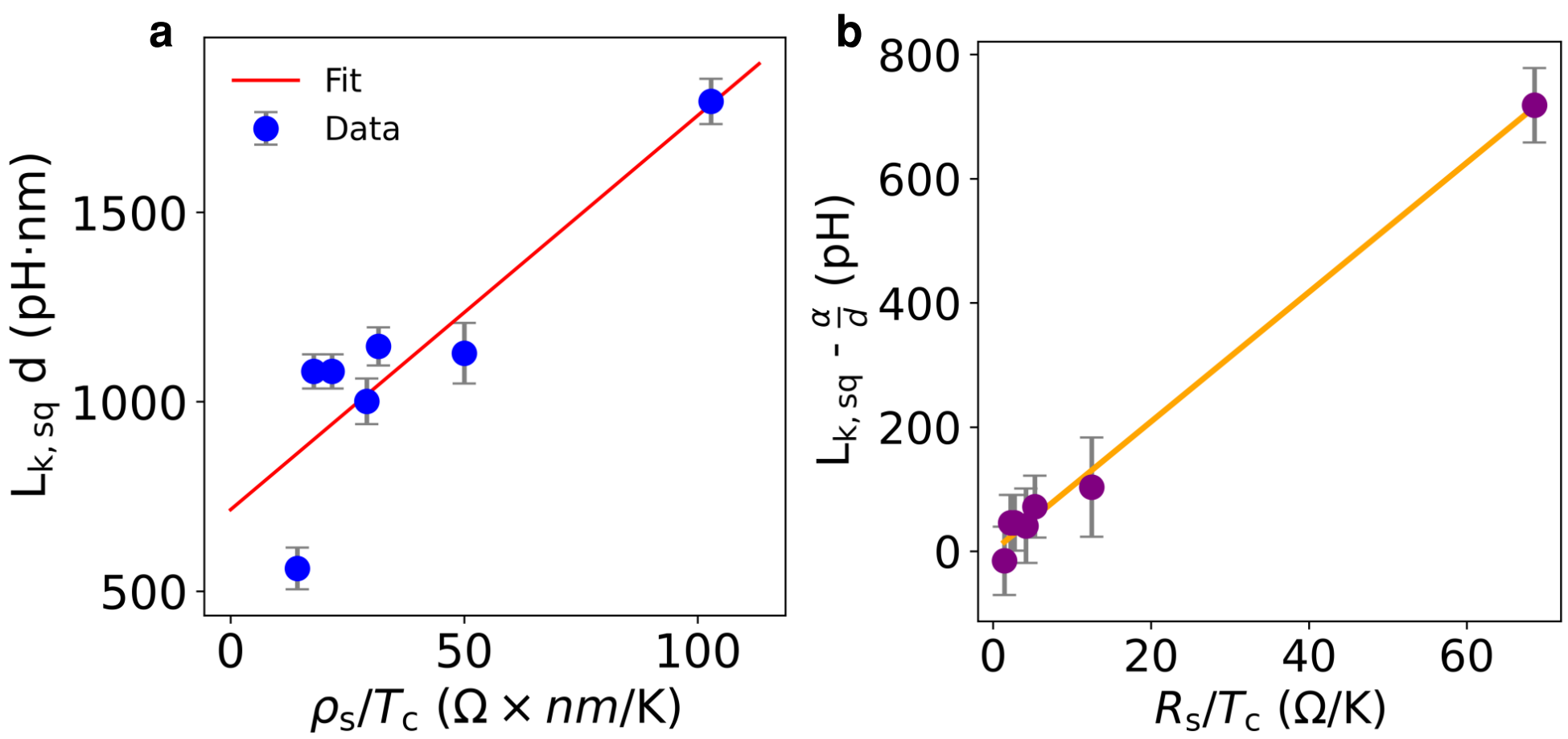

Supplementary Figure 5: **a,** The linear relationship of $L_{\mathrm{k,sq}} \times d$ with $\rho_{\mathrm{s}}/T_{\mathrm{c}}$ where the intercept of the y-axis sets the value of the fitting parameter $\alpha = \frac{m_{eff}}{n_0 e^2}$. **b,** $L_{\mathrm{k,sq}} - \frac{\alpha}{d}$ is plotted as a function of $R_{\mathrm{s}}/T_{\mathrm{c}}$, where the linear fit shows the dirty limit contribution for the thinner samples of $NbSe_2$. The error bars arise from the propagated fit uncertainty in the resonance frequencies extracted from circle fits to the complex S_{21} data.

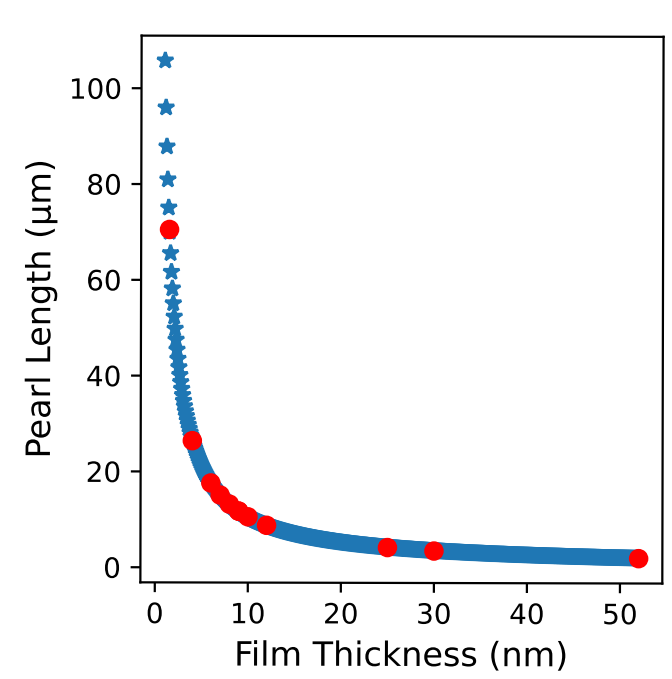

Supplementary Figure 6: **Pearl length estimation (blue) of $NbSe_2$ of different thicknessess with consideration of λ_L = 230 nm.** The $NbSe_2$ samples of this work are identified in red circles.

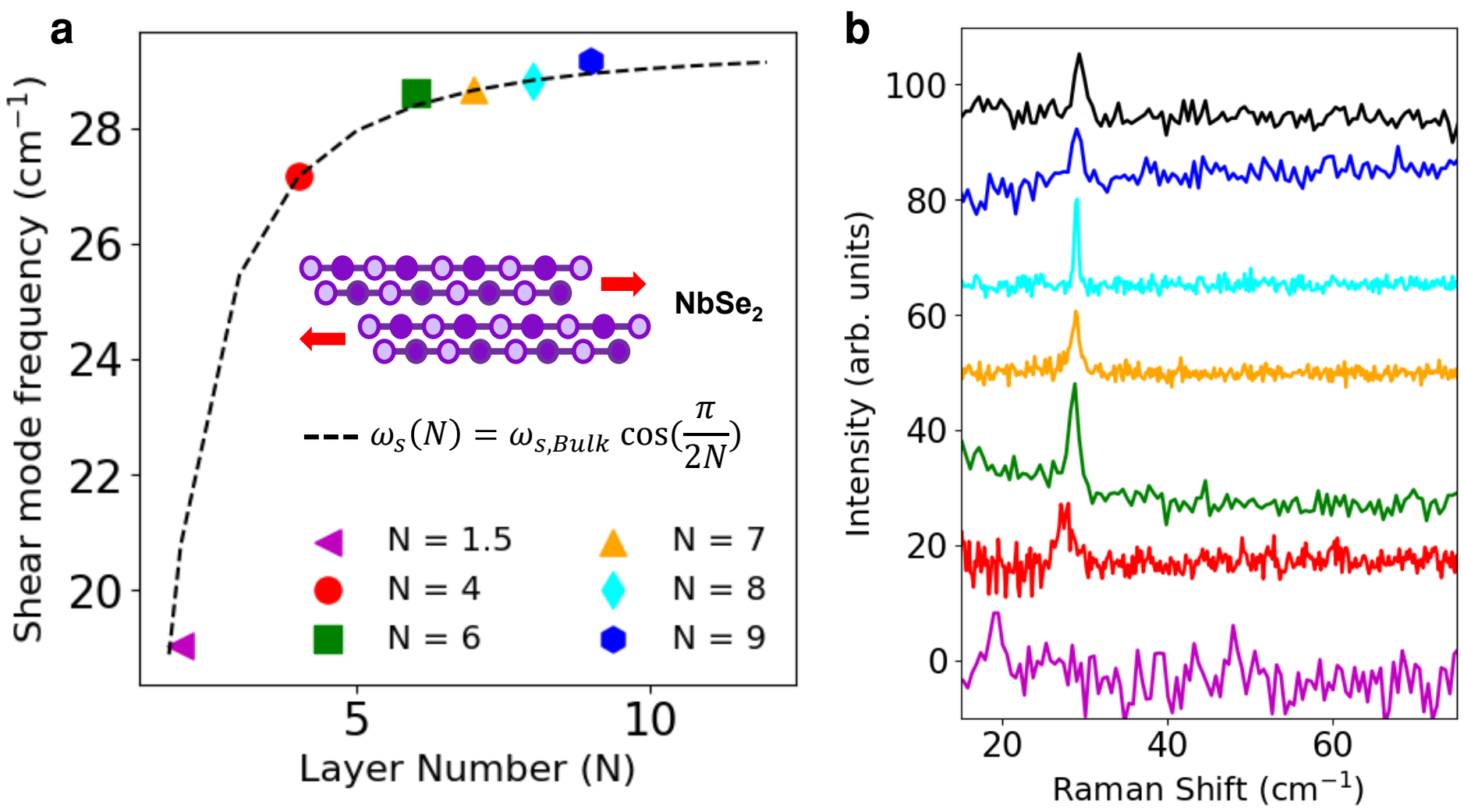

Supplementary Figure 7: **Low-frequency Raman spectroscopy on different layers of $NbSe_2$ samples at room temperature under vacuum for the perpendicular polarization configuration. a,** Plot of shear mode freqeuency versus layer numbers of $NbSe_2$. The dotted blue line shows the $\omega_s = \omega_{s,Bulk}\cos\left(\frac{\pi}{2N}\right)$ relation with $\omega_{s,Bulk} = 29.39\,\text{cm}^{-1}$. **b,** Low-frequency Raman spectra of $NbSe_2$ with layer number N = 4 – 8 and bulk $NbSe_2$. For clarity, the spectra are vertically displaced.

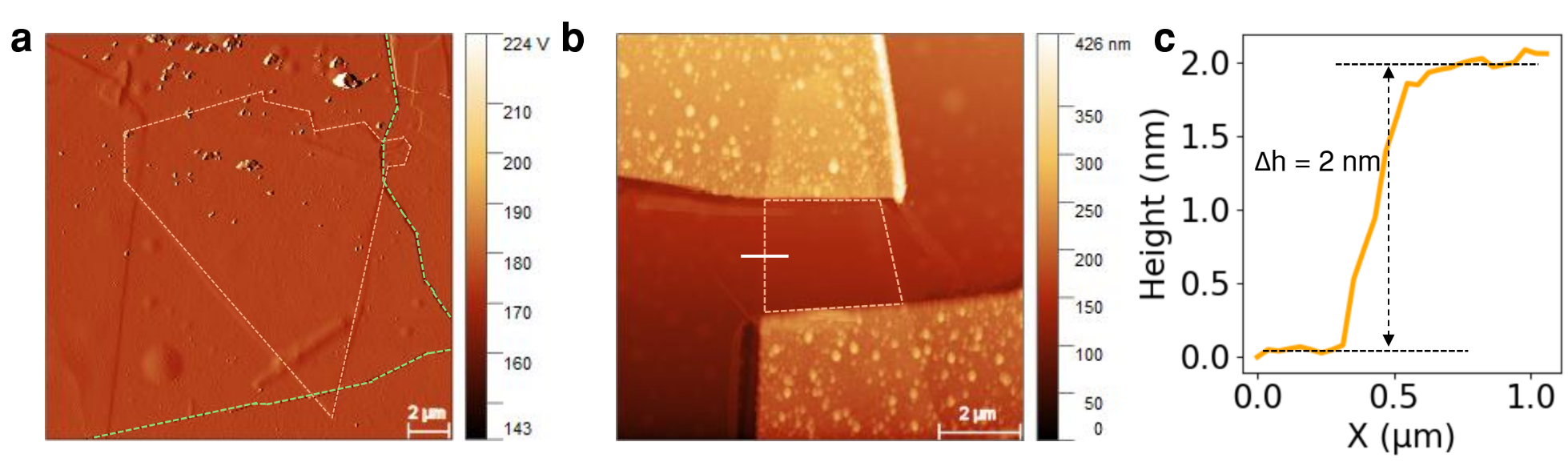

Supplementary Figure 8: **AFM topographic image of hBN encapsulated $NbSe_2$ devices. a,** Amplitude topography of the $NbSe_2$ flake following the assembly of the hBN-$NbSe_2$-hBN heterostructures (hBN-green and $NbSe_2$-orange). **b,** Height profile along the indicated path (white line) to determine the thickness of $NbSe_2$.
