# Supplementary material for: Kinetic inductance of few-layer NbSe2 in the two-dimensional limit
Source: Nat Commun. 2026 Jul 23;17:9007. doi: 10.1038/s41467-026-75672-8 (PMC13507152; doi:10.1038/s41467-026-75672-8)
Supplement: Supplementary file 2 [file 41467_2026_75672_MOESM2_ESM.pdf]

nature portfolio

Peer Review File

Kinetic Inductance of Few-Layer NbSe<2sub> in the Two-Dimensional Limit

Corresponding Author: Ms Sameia Zaman

This file contains all reviewer reports in order by version, followed by all author rebuttals in order by version.

Version 0:

Reviewer comments:

Reviewer #1

(Remarks to the Author)
The authors presented a measurement of the thickness dependence of the kinetic inductance in 2D NbSe2 using superconducting resonators. They find a large inductance in the atomically thin limit and also a crossover from clean to dirty limit as the sample thickness reduces. They further characterized the Kerr nonlinear response of the device and found that the nonlinearity is insignificant compared to other large kinetic inductance superconductors.

The work is interesting and timely. It combines superconducting resonator circuitry with 2D superconductors for quantitative measurements of the kinetic inductance. I support its publication in nature communications after the authors address my question.

I only have one question, which confused me. I am confused by the data in Fig. 4 and equation 2. Based on Fig. 4a, the experimental data is pretty well described by a 1/d dependence, which in turn would mean that the normal state resistivity rho_s is nearly independent of d. Then, there should not be a crossover from clean to dirty limit as the sample thickness reduces. On the other hand, the data in Fig. 4c and 4d suggest the presence of such crossover. And I presume that the 4 data points are from samples with different thicknesses. Then, the clean limit value according to equation 2 should be different for each data point because of the d dependence. Is the dependence in fig. 4d on RS/Tc solely from the dependence of RS on d? But in that case, there should be no crossover from clean to dirty. Or are the 4 data points from samples of nearly the same thickness but varying disorder? I am just confused by the discussions there. I hope that the authors can clarify and help me understand.

Reviewer #2

(Remarks to the Author)
In the manuscript titled 'Kinetic Inductance of Few-Layer NbSe2 in the Two-Dimensional Limit', the authors report measurements of the kinetic inductance of NbSe2 at microwave frequencies by employing cQED techniques.

The kinetic indictance is estimated by comparing the shift in the resonance frequency of a lambda/4 coplanar waveguide Al-resonator terminated with NbSe2 to a similar Al-terminated reaonator.
They report L_{k,sq} for multiple devices with varying NbSe2 thickness and claim a crossover from the clean to the dirty limit of superconductivity as NbSe2 thickness decreases.

The devices are state-of-the-art and the paper is well written.

I believe the techniques and the results will be of interest to experts in the field. However, I have a few comments and questions that must be addressed before evaluating the claims in their entirety.

1) The authors rely on the resonance frequency shifts in NbSe2 resonators compared to the control resonators with a similar geometry. However, the authors do not provide the reader insights into how reliable this comparison is.

2) What is the uncertainty in the resonance frequency of the control resonators? Did the authors study various control resonators with the same dimensions and compare their resonance frequency. What variance should one expect in the resonance frequency for resonators with the same design?

3) If not via experiments, do their simulations across various designs provide an insight into the variance in resonance frequency?

4) What is the impact of the contacts, both Al-contacts to NbSe2 and the bridging contacts between Al, on the resonance frequency?

5) What is the impact of adding dc contacts and LC filters on the resonance frequency?

6) The authors apply a dc current which increases the kinetic inductance quadratically as expected. Can the authors measure the critical current via dc measurements, use this to estimate I*. And extract kinetic inductance from the fitting? How does it match with the kinetic inductance estimated from the frequency shift?

6) Line 176: It is not clear why the authors state that the '...quadratic dependence on the DC bias current (Fig. 3f), [is] consistent with the anisotropic
nodeless pairing of NbSe2 reported in previous studies [43, 60].'
This holds true in general in the Ginzburg-Landau framework as the authors state just before and anisotropic gap should have no impact on this dependence. If the authors' emphasis is on a nodeless gap, please clarify for the reader.

5) Line 126: The authors write 'To achieve a highly transparent, superconducting contact, we make [...] followed by in-situ argon ion milling and aluminum deposition'
And they list the milling parameters in Table S1.

Furthermore they write.
Line 502: 'We chose the optimum ion milling time of 105 seconds...'
However, from the information they provide it is not clear if milling has a systematic impact on contact resistance. Except for a milling time of 80 s, where the contact resistance is large, other milling times seem to have no impact.
It is not clear why 105 s is the optimal time.

The authors should clarify the impact of no Ar milling on contact resistance? Or if they tried much longer milling times?

6) The text unfortunately doesn't cite some key references.
One crucial example is [6a] M. Kriedel et al. PhysRevResearch 6, 043245 (2024) where the authors measured the kinetic inductance of NbSe2, using the same techniques - integrating it into a microwave resonator.

Furthermore, often the reader gets an impression that these are the first microwave measurements in materials other than in amorphous superconducting thin films.

Line 59: 'Recent efforts toward this goal have focused on implementing superinductors using amorphous superconducting thin films'

Line 187: 'Notably, NbSe2, particularly in the few-monolayer regime, exhibits one of the highest sheet kinetic inductances reported at comparable thicknesses, surpassing those of many conventional high-impedance superconductors.'

Line 241: '...reaches 1.2 nH/sq at monolayer thickness — among the highest reported for superconductors within this thickness regime.'

This is not true. The authors own works have measured this in graphene based superconductors. And other van der Waals superconductors have been probed.
[6b] Tanaka, M. et al. Nature 638, 99–105 (2025).
[6c] Banerjee, A. et al. Nature 638, 93–98 (2025).
[6d] Jin, H. et al. Nano Lett. 2025, 25, 8, 3191–3198 (2025).
[6e] Jha, R. et al. Phys. Rev. Lett. 134, 216001 (2025).
[6f] Tian, H. et al. Nature 614, 440–444 (2023).

I suggest that the authors provide the reader a comprehensive overview of the relevant references.

7) How is the error in L_{k,sq}, error bars in Fig. 4a , estimated?

8) There's a typo in equation 27, in the supplementary information.

In summary, the authors should clearly discuss the reliability of the comparison with the control resonator and the variance expected in coplanar waveguide resonator designs. And they should give the reader a better overview of similar or related works.

Version 1:

Reviewer comments:

Reviewer #1

(Remarks to the Author)
The authors had addressed my question. I support its publication.

Reviewer #2

(Remarks to the Author)
The authors have provided sufficient information to support their claims. I support the publication of the article in Nature Communications.

Open Access This Peer Review File is licensed under a Creative Commons Attribution 4.0 International License, which permits use, sharing, adaptation, distribution and reproduction in any medium or format, as long as you give appropriate credit to the original author(s) and the source, provide a link to the Creative Commons license, and indicate if changes were made.
In cases where reviewers are anonymous, credit should be given to 'Anonymous Referee' and the source.
The images or other third party material in this Peer Review File are included in the article's Creative Commons license, unless indicated otherwise in a credit line to the material. If material is not included in the article's Creative Commons license and your intended use is not permitted by statutory regulation or exceeds the permitted use, you will need to obtain permission directly from the copyright holder.
To view a copy of this license, visit https://creativecommons.org/licenses/by/4.0/

Response

Reviewer comment: black

Response: blue

Corresponding change in the manuscript: red

Reviewer 1

The authors presented a measurement of the thickness dependence of the kinetic inductance in 2D $NbSe_2$ using superconducting resonators. They find a large inductance in the atomically thin limit and also a crossover from clean to dirty limit as the sample thickness reduces. They further characterized the Kerr nonlinear response of the device and found that the nonlinearity is insignificant compared to other large kinetic inductance superconductors.

The work is interesting and timely. It combines superconducting resonator circuitry with 2D superconductors for quantitative measurements of the kinetic inductance. I support its publication in Nature Communications after the authors address my question.

I only have one question, which confused me. I am confused by the data in Fig. 4 and equation 2. Based on Fig. 4a, the experimental data is pretty well described by a 1/d dependence, which in turn would mean that the normal state resistivity ρ_s is nearly independent of d. Then, there should not be a crossover from clean to dirty limit as the sample thickness reduces. On the other hand, the data in Fig. 4c and 4d suggest the presence of such crossover. And I presume that the 4 data points are from samples with different thicknesses. Then, the clean limit value according to equation 2 should be different for each data point because of the d dependence. Is the dependence in fig. 4d on R_s/T_c solely from the dependence of R_s on d? But in that case, there should be no crossover from clean to dirty. Or are the 4 data points from samples of nearly the same thickness but varying disorder? I am just confused by the discussions there. I hope that the authors can clarify and help me understand.

We thank the reviewer for the positive comments and for recognizing the importance and broad interest of our work to the readership of Nature Communications. The reviewer raised an important question about the clean- to dirty-limit crossover that we reported, to which we would like to respond below.

We proposed Eq. 2 in the main text to describe our measurement results. Within the Ginzburg–Landau (GL) framework, this model accounts for both the kinetic inductance determined by the phenomenological parameter λ_L (first term) and the contribution arising from scattering processes (second term). In the dirty

superconductor regime, where the BCS coherence length ξ_0 exceeds the mean free path l_{mfp} of the charge carriers, scattering of the electrons forming Cooper pairs becomes significant and contributes substantially to the total kinetic inductance. In contrast, in the clean limit ($\xi_0 < l_{\mathrm{mfp}}$), Cooper pairs can be regarded as particles that propagate ballistically across the sample (see Sec. S7.3 of the Supplementary Information). In this model, the sheet kinetic inductance of a measured device contains contributions from both the clean-limit term (first term) and the dirty-limit term (2nd term), with each scaling linearly with $1/d$. We can rewrite Eq. 2 as

$$L_{\mathrm{k,sq}} = \frac{1}{d}\left(\frac{m_{\mathrm{eff}}}{n_0 e^2} + \frac{\hbar}{1.764 k_{\mathrm{B}}}\frac{\rho_{\mathrm{s}}}{T_{\mathrm{c}}}\right) = \frac{\alpha}{d} + \beta\frac{R_{\mathrm{s}}}{T_{\mathrm{c}}} \tag{1}$$

Samples D1-D9 in Table 1 (plotted in Fig. 4a) cover a large range of $L_{\mathrm{k,sq}}$ and exhibit $1/d$ dependence via the first term in Eq. 1. However, there can still be a weak thickness dependence in α ($\frac{m_{\mathrm{eff}}}{n_0 e^2}$) and in the $\frac{\hbar}{1.764 k_{\mathrm{B}}}\frac{\rho_{\mathrm{s}}}{T_{\mathrm{c}}}$-term, especially for the thicker samples, which cluster near the bottom of Fig. 4c (main text). Figure 4c-d explicitly plots $L_{\mathrm{k,sq}}$ versus $R_{\mathrm{s}}/T_{\mathrm{c}}$ for the seven data points listed in Table 1. The plot captures the combined effects of thickness and scattering processes. Note that the variation in R_s/T_c across devices of different thickness is not determined by the thickness alone, but also likely reflects sample-to-sample variations in disorder (e.g., impurity scattering, interfaces, and fabrication-induced inhomogeneity) as well as differences in the superconducting gap, both of which affect ρ and T_{c}. Therefore, the analysis in Fig. 4c-d incorporates contributions from both thickness variation and disorder in the samples. The four points (marked in red) are close in thickness, indicating a weak 'd' dependence, and the change in R_s/T_c is dominant. As a result, a linear dependence of $L_{\mathrm{k,sq}}$ on R_s/T_c, with a finite intercept as it is observed in our data, reflects the coexistence of clean- and dirty-limit contributions, consistent with this model. The positive y-intercept corresponds to the clean-limit term α/d, while the slope captures the contribution from scattering in the dirty-superconductor regime (see Sec. S7.3 of the Supplementary Information).

However, what we referred to as the "clean limit" in Fig. 4c-d was a dotted line near the lowest α/d value. We agree this might not be the best representation and have hence plotted (main text: Fig. 4c-d) the clean-limit contribution of each data point (marked with purple hexagons) in the revised main text. We observe that α/d is very close to the measured $L_{k,sq}$ for thicker samples (10 nm), whereas for thin samples the dirty-limit contribution dominates. From the revised Fig. 4c-d, one observes a monotonic increase in the ratio $L_{\mathrm{k,sq}}/L_{\mathrm{k,sq,clean}}$ as the thickness decreases, indicating that the dirty-limit contribution becomes significant. This may be attributed to enhanced disorder in thinner devices, where the carriers are more strongly influenced by interfaces, encapsulated layers, and surface scattering. Additional contributions from multi-band superconductivity in $NbSe_2$, where the superconducting gap evolves with thickness [1] may further enhance the coexistence of clean-and dirty-limit behavior. In summary, Fig. 4a captures the dominant $1/d$

scaling, while Figs. 4c and 4d indicate disorder-driven variations of kinetic inductance with the thickness of $NbSe_2$ samples.

The revised text reads: "Figure 4c plots the measured sheet kinetic inductance $L_{\mathrm{k,sq}}$ and clean-limit contribution (the first term of equation 2) as a function of $R_{\mathrm{s}}/T_{\mathrm{c}}$. The R_{s} and T_{c} values are obtained from both our transport measurements and previous studies [41, 42] (shown in green and black circles in Fig. 4c and summarized in Table S3). We estimate α $\left(= \frac{m_{\mathrm{eff}}}{n_0 e^2}\right)$ for the clean-limit contribution, assuming α is independent of disorder and film thickness (see Supplementary Information Fig. S5). We observe a linear dependence for the larger values of $L_{\mathrm{k,sq}}$ in Fig. 4c. A linear fit yields a slope $\beta = 16.3$, which captures the dirty-limit contribution. In contrast, the α/d term for each data point reflects the clean-limit component, which approaches the total $L_{\mathrm{k,sq}}$ in thicker samples."

Layers	$L_{\mathrm{k,sq}}$ (pH/sq)	DC transport (independent devices)		
	MW device ID	T_{c} (K)	R_{s} (Ω/sq)	$R_{\mathrm{s}}/T_{\mathrm{c}}$ (Ω/K)
1	1195 (D1)	3.5[2]	240[2]	68.57
4	282 (D2)	4.8	60	12.5
6	191 (D3)	5.5	29	5.27
7	143 (D4)	6.0	25	4.17
8	135 (D5)	7.0	19	2.71
8	135 (D5)	6.3[3]	14[3]	2.22
10	56 (D9)	7.0[2]	10[2]	1.43

Table 1: **Summary of devices used for microwave measurements and DC transport measurement.** $L_{\mathrm{k,sq}}$ is extracted from microwave resonator measurements, while T_{c} and R_{s} are obtained from independent DC transport devices of similar layer number or from literature.

Reviewer 2

In the manuscript titled "Kinetic Inductance of Few-Layer $NbSe_2$ in the Two-Dimensional Limit", the authors report measurements of the kinetic inductance of $NbSe_2$ at microwave frequencies by employing cQED techniques.

The kinetic inductance is estimated by comparing the shift in the resonance frequency of a $\lambda/4$ coplanar waveguide Al-resonator terminated with $NbSe_2$ to a similar Al-terminated resonator. They report $L_{k,sq}$ for multiple devices with varying $NbSe_2$ thickness and claim a crossover from the clean to the dirty limit of superconductivity as $NbSe_2$ thickness decreases.

The devices are state-of-the-art, and the paper is well written.

We thank the reviewer for detailed comments and suggestions. The effort and time the reviewer has taken

to review our manuscript is deeply appreciated. We thank the reviewer for emphasizing a critical aspect of the technique we used for this study. The reviewer raised some constructive concerns, to which we would like to respond point-by-point below.

I believe the techniques and the results will be of interest to experts in the field. However, I have a few comments and questions that must be addressed before evaluating the claims in their entirety.

1. The authors rely on the resonance frequency shifts in $NbSe_2$ resonators compared to the control resonators with a similar geometry. However, the authors do not provide the reader with insights into how reliable this comparison is.

   We thank the reviewer for raising this important point regarding the reliability of comparing the resonance frequency shifts of the $NbSe_2$ resonators to control resonators with similar geometry. To ensure that the extracted frequency shifts reflect intrinsic material properties rather than geometric variations, we designed the control resonators - not to be confused with the "spectator" resonator on the same chip - to be nominally identical in geometry (length, width, coupling capacitance, and ground configuration) and fabricated them within the same processing run. This approach minimizes systematic variations arising from lithography, film thickness nonuniformity, and fabrication conditions. Additionally, the measured devices had a similar chip layout and were measured using the same experimental setup. We also emphasize that the measured resonance frequencies of five control resonators with nominally identical geometry have a standard deviation of 21.65 MHz (Fig. 1), which is smaller than the frequency shift attributed to the kinetic inductance of $NbSe_2$ (Table 1 main text).

2. What is the uncertainty in the resonance frequency of the control resonators? Did the authors study various control resonators with the same dimensions and compare their resonance frequency? What variance should one expect in the resonance frequency for resonators with the same design?
   The uncertainty in the resonance frequency of the control resonators primarily arises from fabrication-induced variations. In our process, these variations originate from two main stages:

   (a) Maskless photolithography followed by wet etching of aluminum thin films can introduce small variations in resonator linewidth, length, and capacitor geometry, leading to device-to-device frequency scatter. To quantify this effect, we measured the resonance frequencies of 12 *spectator* resonators across the chips used in this experiment (Table 1, main text). We find a mean resonance frequency of 6.066 GHz with a standard deviation of 5.74 MHz, corresponding to a relative variation of $\sim 0.095\%$.

   (b) Additional variability can arise from e-beam lithography, metal deposition, and lift-off used for

contacted and/or device-specific structures, which may modify the effective circuit parameters. To assess this contribution, we fabricated nominally identical Al-terminated *experimental* resonators with the same geometry as device D3 and compared their resonance frequencies. Across five such resonators, we obtain a mean resonance frequency of 5.318 GHz with a standard deviation of 21.65 MHz (relative variation $\sim 0.41\%$). Importantly, this variation remains significantly smaller than the observed frequency shift of ~ 450 MHz associated with kinetic inductance.

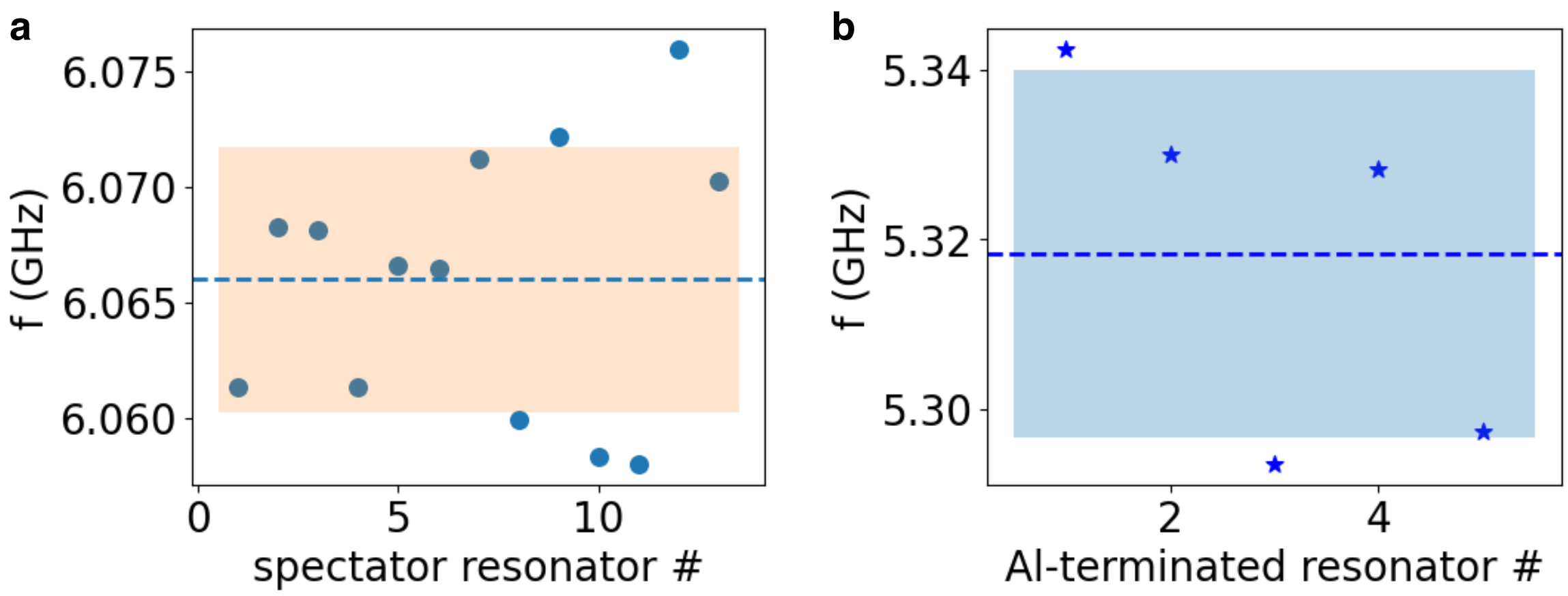

Figure 1: **Experimentally measured resonant frequencies of a, 12 *spectator* resonators exhibit a mean resonant frequency of 6.066 GHz (dashed line), with a standard deviation of 5.74 MHz, and b, 5 Al-terminated *experimental* resonators with nominally identical geometry as device D3. The distribution has a mean resonant frequency of 5.318 GHz (dashed line) with a standard deviation of 21.65 MHz.**

These measurements establish the expected device-to-device variation in resonance frequency for nominally identical designs. We note that for devices with a substantial kinetic inductance contribution (D1–D10), the induced frequency shift is much larger than the fabrication-induced spread, enabling the reliable extraction of kinetic inductance. In contrast, for devices with smaller kinetic inductance contribution (e.g., D11 and D12), the corresponding frequency shifts become comparable to the intrinsic fabrication variance, limiting the precision of the extracted values.

3. If not via experiments, do their simulations across various designs provide an insight into the variance in resonance frequency?

To quantify the variation in the control resonator frequency, we fabricated five Al-terminated control resonators with nominally identical geometry to device D3. Across these five devices, we obtain a mean resonance frequency of 5.318 GHz with a standard deviation of 21.65 MHz (relative variation

$\sim 0.41\%$). We note that for devices with a substantial kinetic inductance contribution (D1–D10), the induced frequency shift is much larger than the fabrication-induced spread, enabling the reliable extraction of kinetic inductance. In contrast, for devices with smaller kinetic inductance contribution (e.g., D11 and D12), the corresponding frequency shifts become comparable to the intrinsic fabrication variance, limiting the precision of the extracted values.

4. What is the impact of the contacts, both Al-contacts to $NbSe_2$ and the bridging contacts between Al, on the resonance frequency?

   The contact resistances from Al-contacts to $NbSe_2$ and the bridging contacts between Al will act as a series resistance to the $\lambda/4$ resonator. A series resistance would decrease the internal quality factor Q_i for the $NbSe_2$-terminated resonator, but does not affect the resonant frequency. This is also supported by our simulation shown in Fig. 2. We use the SONNET finite-element solver to determine the resonant frequency of the coplanar waveguide resonator. We model the Al film as a two-dimensional perfect electrical conductor on a 350 μm silicon (Si) substrate, with 500 μm of vacuum above the film, and calculate the S-parameters at the microwave ports.

   The lumped-element model for a $\lambda/4$ resonator terminated to ground with a resistance R = 0.0, 0.1, 0.2, 0.3, 0.4, and 0.5 Ω representing the contact resistance in series with the superconducting device. The simulation illustrates that while the resonant frequency remains essentially unchanged, the internal quality factor Q_i is sensitive to the series resistance, as it decreases rapidly with even a small resistance of 0.5 Ω.

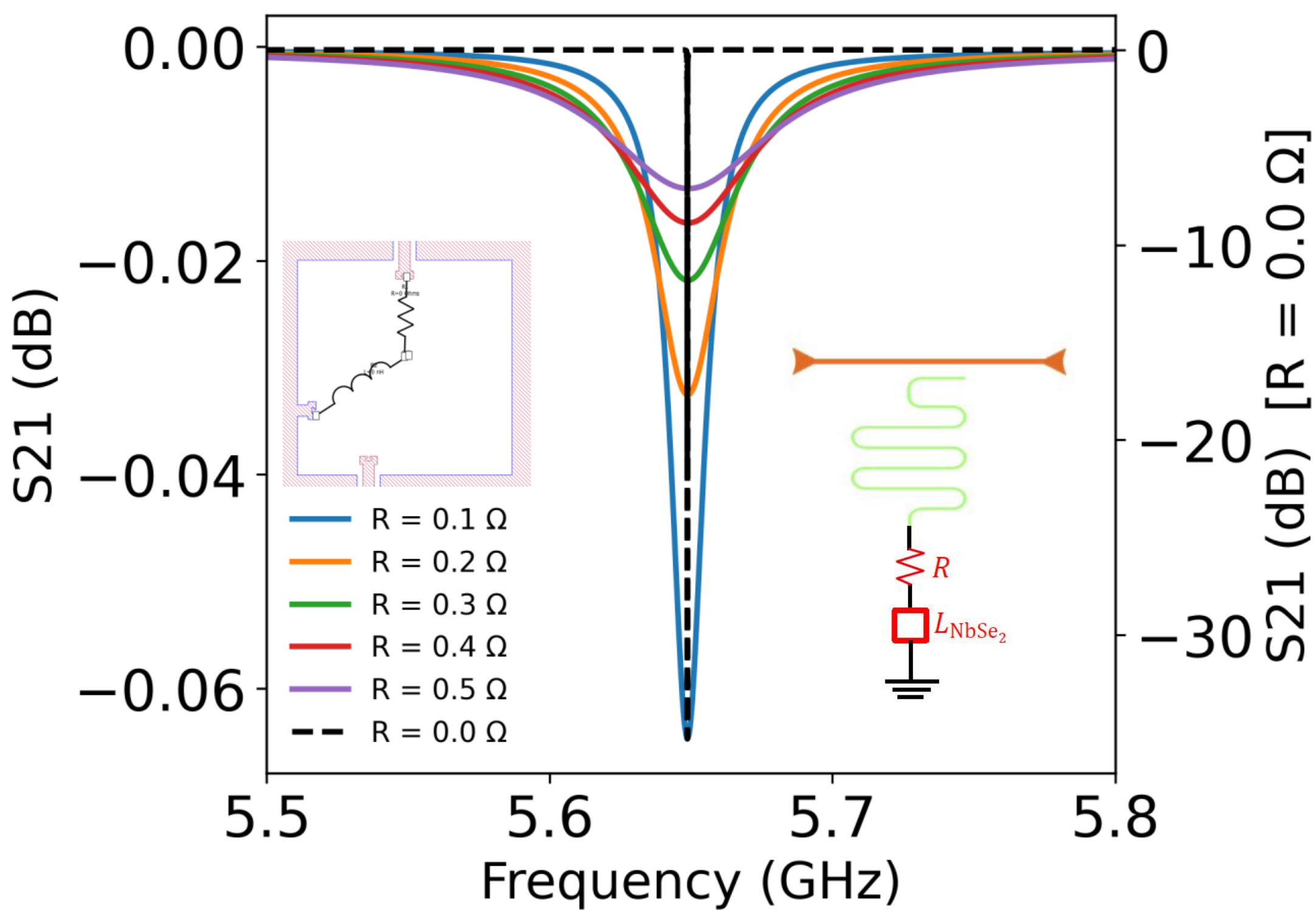

Figure 2: **Microwave simulation of superconducting $\lambda/4$ resonators terminated with a series resistance R. The resistance R has been swept from 0 Ω to 0.1 Ω in the lump-element model (shown in the insets). The resonance frequency shows a negligible change with R.**

5. What is the impact of adding dc contacts and LC filters on the resonance frequency?

We thank the reviewer for pointing out a feature for further discussion. To address this, we are considering two scenarios:

- **Presence of filters without connections**: We simulated $\lambda/2$ experimental resonators both with and without LC filters on the chip, observing no impact on resonator frequency. Figure 3a shows the microwave simulations with up to three filters added, demonstrating that the change in resonant frequency is negligible. The chips listed in Table 1 (main text) have on-chip filters, and only devices D6 and D10 had one DC electrode connected to the sample.
- **Filter connected to the resonator**: We simulated the $\lambda/4$ experimental resonator with and without a DC line and filters connected to its terminated end (Fig. 3b). We observed a frequency shift of 4.1 MHz between the two configurations. This shift likely originates from the change

in impedance caused by the addition of the DC line and the LC filter. We emphasize that this shift has been accounted for by utilizing the all-aluminum control resonator (Device ID D6 and D10) with an identical configuration to the sample resonator, which effectively accounts for these geometric effects. In addition, the shift caused by adding DC electrodes and LC filters is smaller than the shift caused by $NbSe_2$ (ranging from 20 MHz to 1.68 GHz).

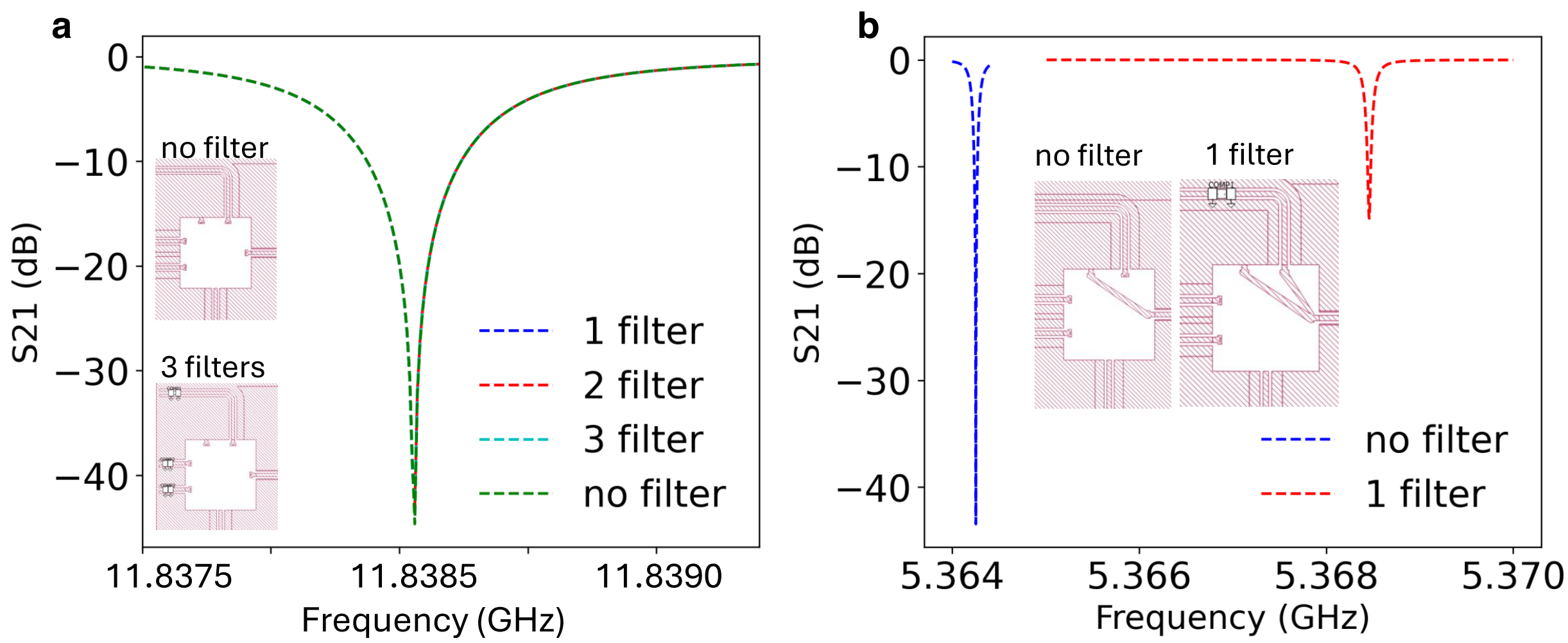

Figure 3: **Microwave simulations of superconducting resonators. a, $\lambda/2$ resonators with on-chip filters. b, $\lambda/4$ resonators with and without an electrode connection through a filter.**

6. The authors apply a dc current which increases the kinetic inductance quadratically as expected. Can the authors measure the critical current via dc measurements, use this to estimate I^*. And extract the kinetic inductance from the fitting? How does it match with the kinetic inductance estimated from the frequency shift?

   We thank the reviewer for this insightful suggestion. While DC transport measurements were not performed on this specific device due to the device's 2-probe configuration and the series Al elements (LC filter), we did estimate the critical current of another device of 8 nm thickness (close to the device reported in Fig 3 main text) in Fig. S1 (e-f), which yielded a critical current density $J_c = 2 \times 10^{10}$ A/m^2. This corresponds to a critical current of approximately $I_c \approx 0.7$ mA for our device dimensions. This value is comparable to the characteristic current $I^* \approx (0.4$ mA) extracted from the microwave measurement, indicating reasonable agreement (within the same order of magnitude). This agreement between the DC and microwave results supports the conclusions that the observed non-linearity arises from the intrinsic kinetic inductance of the $NbSe_2$ flake.

   Line 176: It is not clear why the authors state that the '...quadratic dependence on the DC bias current

(Fig. 3f), [is] consistent with the anisotropic nodeless pairing of $NbSe_2$ reported in previous studies [43, 60].' This holds true in general in the Ginzburg-Landau framework as the authors state just before and anisotropic gap should have no impact on this dependence. If the authors' emphasis is on a nodeless gap, please clarify for the reader.

We thank the reviewer for pointing out this lack of clarity. The quadratic dependence of the resonance frequency shift on the applied dc bias current follows directly from the Ginzburg–Landau description of the nonlinear kinetic inductance. Within this framework, the superfluid density decreases quadratically with current, leading to a corresponding quadratic increase in kinetic inductance and hence a quadratic shift of the resonance frequency.

Our intention in referring to the anisotropic nodeless pairing reported for $NbSe_2$ in Refs. [43,60] was not to suggest that the anisotropy of the superconducting gap is responsible for this quadratic dependence. Rather, the key point is that $NbSe_2$ is known to host a fully gapped (nodeless) superconducting state, albeit with moderate gap anisotropy. In such systems, the nonlinear kinetic inductance is expected to follow the standard Ginzburg–Landau behavior at low currents, producing the observed quadratic dependence. To avoid confusion, we have revised the text (main text: line 177-178) to clarify that the quadratic current dependence arises from the general Ginzburg–Landau description of superconducting kinetic inductance, and that our observation is consistent with the expected behavior of a fully gapped superconducting state in $NbSe_2$, rather than being a direct signature of gap anisotropy.

The revised text reads: "We observe that both the resonant frequency and the kinetic inductance per square exhibit a quadratic dependence on the DC bias current (Fig. 3f), consistent with the fully gapped pairing of $NbSe_2$ reported in previous studies [46, 63]"

Line 126: The authors write 'To achieve a highly transparent, superconducting contact, we make [...] followed by in-situ argon ion milling and aluminum deposition'. And they list the milling parameters in Table S1. Furthermore, they write. Line 502: 'We chose the optimum ion milling time of 105 seconds...' However, from the information they provide it is not clear if milling has a systematic impact on contact resistance. Except for a milling time of 80 s, where the contact resistance is large, other milling times seem to have no impact. It is not clear why 105 s is the optimal time.

The authors should clarify the impact of no Ar milling on contact resistance? Or if they tried much longer milling times?

We thank the reviewer for pointing out this lack of clarity. We agree that the term "optimum" is misleading, as the contact resistance (R_c) remains consistently low across a range of milling times from

60s to 120s.

Defining the Choice of 105 s: The duration of 105 s was chosen as a standardized, reliable condition within the stable regime observed in Table S1. While 60 s and 90 s also yielded low R_c, 105 s was selected to ensure complete removal of any potential polymer residues or atmospheric contaminants across the entire contact area before Aluminum deposition, providing high reproducibility across multiple device batches.

Impact of "No Milling": Our experiments, consistent with a previous study by [4], show that omitting the Ar milling step entirely resulted in inconsistent contacts with significantly higher resistances. This is attributed to the rapid formation of a native oxide layer on $NbSe_2$. The data in Table S1 demonstrates that milling is necessary to "break" through this oxide layer.

Longer Milling Times: We tested ion-milling times up to 120 s, beyond 90 s, we observed no further reduction in R_c (staying at 0.01 Ω/μm). We did not pursue significantly longer times to avoid over-etching the thin 2D flake ($d \ll 10$ nm), as excessive ion bombardment can degrade the sample and suppress the local T_c.

We have revised the text (Line 126 and Line 516) to replace "optimum" with "chosen reproducible condition" to better reflect that 105 s is within a broad, functional plateau of low contact resistance.

The revised text now reads: "We present DC transport measurements to characterize the contact resistance as a function of ion milling time, with the results summarized in Table S1. We selected 105 s for the ion milling time, which we demonstrated to provide a reproducible and low contact resistance."

7. The text unfortunately doesn't cite some key references. One crucial example is [6a] M. Kriedel et al. PhysRevResearch 6, 043245 (2024) where the authors measured the kinetic inductance of $NbSe_2$, using the same techniques - integrating it into a microwave resonator. Furthermore, often the reader gets an impression that these are the first microwave measurements in materials other than in amorphous superconducting thin films.

Line 59: 'Recent efforts toward this goal have focused on implementing superinductors using amorphous superconducting thin films'

Line 187: 'Notably, $NbSe_2$, particularly in the few-monolayer regime, exhibits one of the highest sheet kinetic inductances reported at comparable thicknesses, surpassing those of many conventional high-impedance superconductors.'

Line 241: '...reaches 1.2 nH/sq at monolayer thickness — among the highest reported for supercon-

ductors within this thickness regime.'

This is not true. The authors own works have measured this in graphene-based superconductors. And other van der Waals superconductors have been probed. [6b] Tanaka, M. et al. Nature 638, 99–105 (2025). [6c] Banerjee, A. et al. Nature 638, 93–98 (2025). [6d] Jin, H. et al. Nano Lett. 2025, 25, 8, 3191–3198 (2025). [6e] Jha, R. et al. Phys. Rev. Lett. 134, 216001 (2025). [6f] Tian, H. et al. Nature 614, 440–444 (2023).

I suggest that the authors provide the reader with a comprehensive overview of the relevant references.

We thank the reviewer for mentioning these important prior demonstrations, and we fully agree they should be acknowledged. We have cited them now on page 2 line 50 (main text).

We have also revised the language in the main text to distinguish the intrinsic superconductors, which are listed in fig 4b, and the voltage-induced superconductor, including MATBG or MATTG.

The revised text reads: "Voltage-induced flat-band superconductors, such as magic-angle twisted bilayer [4, 66] and trilayer graphene [5, 67], exhibit a wide range of inductances depending on bias voltages and are therefore not included in Fig. 4b."

8. How is the error in $L_{k,sq}$, error bars in Fig. 4a estimated?

The error bars in Fig. 4a reflect the geometric uncertainty in the effective number of squares. Because the $NbSe_2$ flakes deviate slightly from an ideal rectangulary geometry, we estimate the uncertainty in the number of squares by comparing the effective rectangular area between the electrodes to the outer flake envelope extracted from microscopy images. The corresponding fractional uncertainty is propagated directly to the error bars.

In addition, we incorporate the fit error from the resonance frequency extraction obtained via circle fitting of the S_{21} data [5, 6]. This contribution is included for both the $NbSe_2$-terminated and the control resonators.

(Error bars are updated in Fig. 4a)

9. There's a typo in equation 27, in the supplementary information.

The typo is corrected.

References

1. Dvir, T. *et al.* Spectroscopy of bulk and few-layer superconducting $NbSe_2$ with van der Waals tunnel junctions. *Nature Communications* **9,** 598 (2018).
2. Khestanova, E. *et al.* Unusual suppression of the superconducting energy gap and critical temperature in atomically thin $NbSe_2$. *Nano Letters* **18,** 2623–2629 (2018).
3. Cao, Y. *et al.* Quality heterostructures from two-dimensional crystals unstable in air by their assembly in inert atmosphere. *Nano Letters* **15,** 4914–4921 (2015).
4. Sinko, M. R. *et al.* Superconducting contact and quantum interference between two-dimensional van der Waals and three-dimensional conventional superconductors. *Physical Review Materials* **5,** 014001 (2021).
5. Probst, S., Song, F., Bushev, P. A., Ustinov, A. V. & Weides, M. Efficient and robust analysis of complex scattering data under noise in microwave resonators. *Review of Scientific Instruments* **86** (2015).
6. Baity, P. G. *et al.* Circle fit optimization for resonator quality factor measurements: Point redistribution for maximal accuracy. *Physical Review Research* **6,** 013329 (2024).

Response

Reviewer comment: black

Response: blue

Corresponding change in the manuscript: red

Round 1

Reviewer 1

The authors presented a measurement of the thickness dependence of the kinetic inductance in 2D $NbSe_2$ using superconducting resonators. They find a large inductance in the atomically thin limit and also a crossover from clean to dirty limit as the sample thickness reduces. They further characterized the Kerr nonlinear response of the device and found that the nonlinearity is insignificant compared to other large kinetic inductance superconductors.

The work is interesting and timely. It combines superconducting resonator circuitry with 2D superconductors for quantitative measurements of the kinetic inductance. I support its publication in Nature Communications after the authors address my question.

I only have one question, which confused me. I am confused by the data in Fig. 4 and equation 2. Based on Fig. 4a, the experimental data is pretty well described by a 1/d dependence, which in turn would mean that the normal state resistivity ρ_s is nearly independent of d. Then, there should not be a crossover from clean to dirty limit as the sample thickness reduces. On the other hand, the data in Fig. 4c and 4d suggest the presence of such crossover. And I presume that the 4 data points are from samples with different thicknesses. Then, the clean limit value according to equation 2 should be different for each data point because of the d dependence. Is the dependence in fig. 4d on R_s/T_c solely from the dependence of R_s on d? But in that case, there should be no crossover from clean to dirty. Or are the 4 data points from samples of nearly the same thickness but varying disorder? I am just confused by the discussions there. I hope that the authors can clarify and help me understand.

We thank the reviewer for the positive comments and for recognizing the importance and broad interest of our work to the readership of Nature Communications. The reviewer raised an important question about the clean- to dirty-limit crossover that we reported, to which we would like to respond below.

We proposed Eq. 2 in the main text to describe our measurement results. Within the Ginzburg–Landau

(GL) framework, this model accounts for both the kinetic inductance determined by the phenomenological parameter λ_L (first term) and the contribution arising from scattering processes (second term). In the dirty superconductor regime, where the BCS coherence length ξ_0 exceeds the mean free path $l_{\rm mfp}$ of the charge carriers, scattering of the electrons forming Cooper pairs becomes significant and contributes substantially to the total kinetic inductance. In contrast, in the clean limit ($\xi_0 < l_{\rm mfp}$), Cooper pairs can be regarded as particles that propagate ballistically across the sample (see Sec. S7.3 of the Supplementary Information). In this model, the sheet kinetic inductance of a measured device contains contributions from both the clean-limit term (first term) and the dirty-limit term (2nd term), with each scaling linearly with $1/d$. We can rewrite Eq. 2 as

$$L_{\rm k,sq} = \frac{1}{d}\left(\frac{m_{\rm eff}}{n_0 e^2} + \frac{\hbar}{1.764 k_{\rm B}}\frac{\rho_{\rm s}}{T_{\rm c}}\right) = \frac{\alpha}{d} + \beta\frac{R_{\rm s}}{T_{\rm c}} \tag{1}$$

Samples D1-D9 in Table 1 (plotted in Fig. 4a) cover a large range of $L_{\rm k,sq}$ and exhibit $1/d$ dependence via the first term in Eq. 1. However, there can still be a weak thickness dependence in α ($\frac{m_{\rm eff}}{n_0 e^2}$) and in the $\frac{\hbar}{1.764 k_{\rm B}}\frac{\rho_{\rm s}}{T_{\rm c}}$-term, especially for the thicker samples, which cluster near the bottom of Fig. 4c (main text). Figure 4c-d explicitly plots $L_{\rm k,sq}$ versus $R_{\rm s}/T_{\rm c}$ for the seven data points listed in Table 1. The plot captures the combined effects of thickness and scattering processes. Note that the variation in R_s/T_c across devices of different thickness is not determined by the thickness alone, but also likely reflects sample-to-sample variations in disorder (e.g., impurity scattering, interfaces, and fabrication-induced inhomogeneity) as well as differences in the superconducting gap, both of which affect ρ and $T_{\rm c}$. Therefore, the analysis in Fig. 4c-d incorporates contributions from both thickness variation and disorder in the samples. The four points (marked in red) are close in thickness, indicating a weak 'd' dependence, and the change in R_s/T_c is dominant. As a result, a linear dependence of $L_{\rm k,sq}$ on R_s/T_c, with a finite intercept as it is observed in our data, reflects the coexistence of clean- and dirty-limit contributions, consistent with this model. The positive y-intercept corresponds to the clean-limit term α/d, while the slope captures the contribution from scattering in the dirty-superconductor regime (see Sec. S7.3 of the Supplementary Information).

However, what we referred to as the "clean limit" in Fig. 4c-d was a dotted line near the lowest α/d value. We agree this might not be the best representation and have hence plotted (main text: Fig. 4c-d) the clean-limit contribution of each data point (marked with purple hexagons) in the revised main text. We observe that α/d is very close to the measured $L_{k,sq}$ for thicker samples (10 nm), whereas for thin samples the dirty-limit contribution dominates. From the revised Fig. 4c-d, one observes a monotonic increase in the ratio $L_{\rm k,sq}/L_{\rm k,sq,clean}$ as the thickness decreases, indicating that the dirty-limit contribution becomes significant. This may be attributed to enhanced disorder in thinner devices, where the carriers are more strongly influenced by interfaces, encapsulated layers, and surface scattering. Additional contributions from

multi-band superconductivity in $NbSe_2$, where the superconducting gap evolves with thickness [1] may further enhance the coexistence of clean-and dirty-limit behavior. In summary, Fig. 4a captures the dominant $1/d$ scaling, while Figs. 4c and 4d indicate disorder-driven variations of kinetic inductance with the thickness of $NbSe_2$ samples.

The revised text reads: "Figure 4c plots the measured sheet kinetic inductance $L_{\mathrm{k,sq}}$ and clean-limit contribution (the first term of equation 2) as a function of R_{s}/T_c. The R_{s} and T_{c} values are obtained from both our transport measurements and previous studies [41, 42] (shown in green and black circles in Fig. 4c and summarized in Table S3). We estimate α $\left(= \frac{m_{\mathrm{eff}}}{n_0 e^2}\right)$ for the clean-limit contribution, assuming α is independent of disorder and film thickness (see Supplementary Information Fig. S5). We observe a linear dependence for the larger values of $L_{\mathrm{k,sq}}$ in Fig. 4c. A linear fit yields a slope $\beta = 16.3$, which captures the dirty-limit contribution. In contrast, the α/d term for each data point reflects the clean-limit component, which approaches the total $L_{\mathrm{k,sq}}$ in thicker samples."

Layers	$L_{\mathrm{k,sq}}$ (pH/sq)	DC transport (independent devices)		
	MW device ID	T_{c} (K)	R_{s} (Ω/sq)	$R_{\mathrm{s}}/T_{\mathrm{c}}$ (Ω/K)
1	1195 (D1)	3.5[2]	240[2]	68.57
4	282 (D2)	4.8	60	12.5
6	191 (D3)	5.5	29	5.27
7	143 (D4)	6.0	25	4.17
8	135 (D5)	7.0	19	2.71
8	135 (D5)	6.3[3]	14[3]	2.22
10	56 (D9)	7.0[2]	10[2]	1.43

Table 1: **Summary of devices used for microwave measurements and DC transport measurement.** $L_{\mathrm{k,sq}}$ is extracted from microwave resonator measurements, while T_{c} and R_{s} are obtained from independent DC transport devices of similar layer number or from literature.

Reviewer 2

In the manuscript titled "Kinetic Inductance of Few-Layer $NbSe_2$ in the Two-Dimensional Limit", the authors report measurements of the kinetic inductance of $NbSe_2$ at microwave frequencies by employing cQED techniques.

The kinetic inductance is estimated by comparing the shift in the resonance frequency of a $\lambda/4$ coplanar waveguide Al-resonator terminated with $NbSe_2$ to a similar Al-terminated resonator. They report $\mathrm{L}_{k,sq}$ for multiple devices with varying $NbSe_2$ thickness and claim a crossover from the clean to the dirty limit of superconductivity as $NbSe_2$ thickness decreases.

The devices are state-of-the-art, and the paper is well written.

We thank the reviewer for detailed comments and suggestions. The effort and time the reviewer has taken to review our manuscript is deeply appreciated. We thank the reviewer for emphasizing a critical aspect of the technique we used for this study. The reviewer raised some constructive concerns, to which we would like to respond point-by-point below.

I believe the techniques and the results will be of interest to experts in the field. However, I have a few comments and questions that must be addressed before evaluating the claims in their entirety.

1. The authors rely on the resonance frequency shifts in $NbSe_2$ resonators compared to the control resonators with a similar geometry. However, the authors do not provide the reader with insights into how reliable this comparison is.

   We thank the reviewer for raising this important point regarding the reliability of comparing the resonance frequency shifts of the $NbSe_2$ resonators to control resonators with similar geometry. To ensure that the extracted frequency shifts reflect intrinsic material properties rather than geometric variations, we designed the control resonators - not to be confused with the "spectator" resonator on the same chip - to be nominally identical in geometry (length, width, coupling capacitance, and ground configuration) and fabricated them within the same processing run. This approach minimizes systematic variations arising from lithography, film thickness nonuniformity, and fabrication conditions. Additionally, the measured devices had a similar chip layout and were measured using the same experimental setup. We also emphasize that the measured resonance frequencies of five control resonators with nominally identical geometry have a standard deviation of 21.65 MHz (Fig. 1), which is smaller than the frequency shift attributed to the kinetic inductance of $NbSe_2$ (Table 1 main text).

2. What is the uncertainty in the resonance frequency of the control resonators? Did the authors study various control resonators with the same dimensions and compare their resonance frequency? What variance should one expect in the resonance frequency for resonators with the same design?
   The uncertainty in the resonance frequency of the control resonators primarily arises from fabrication-induced variations. In our process, these variations originate from two main stages:

   (a) Maskless photolithography followed by wet etching of aluminum thin films can introduce small variations in resonator linewidth, length, and capacitor geometry, leading to device-to-device frequency scatter. To quantify this effect, we measured the resonance frequencies of 12 *spectator* resonators across the chips used in this experiment (Table 1, main text). We find a mean resonance frequency of 6.066 GHz with a standard deviation of 5.74 MHz, corresponding to a relative

variation of $\sim 0.095\%$.

(b) Additional variability can arise from e-beam lithography, metal deposition, and lift-off used for contacted and/or device-specific structures, which may modify the effective circuit parameters. To assess this contribution, we fabricated nominally identical Al-terminated *experimental* resonators with the same geometry as device D3 and compared their resonance frequencies. Across five such resonators, we obtain a mean resonance frequency of 5.318 GHz with a standard deviation of 21.65 MHz (relative variation $\sim 0.41\%$). Importantly, this variation remains significantly smaller than the observed frequency shift of ~ 450 MHz associated with kinetic inductance.

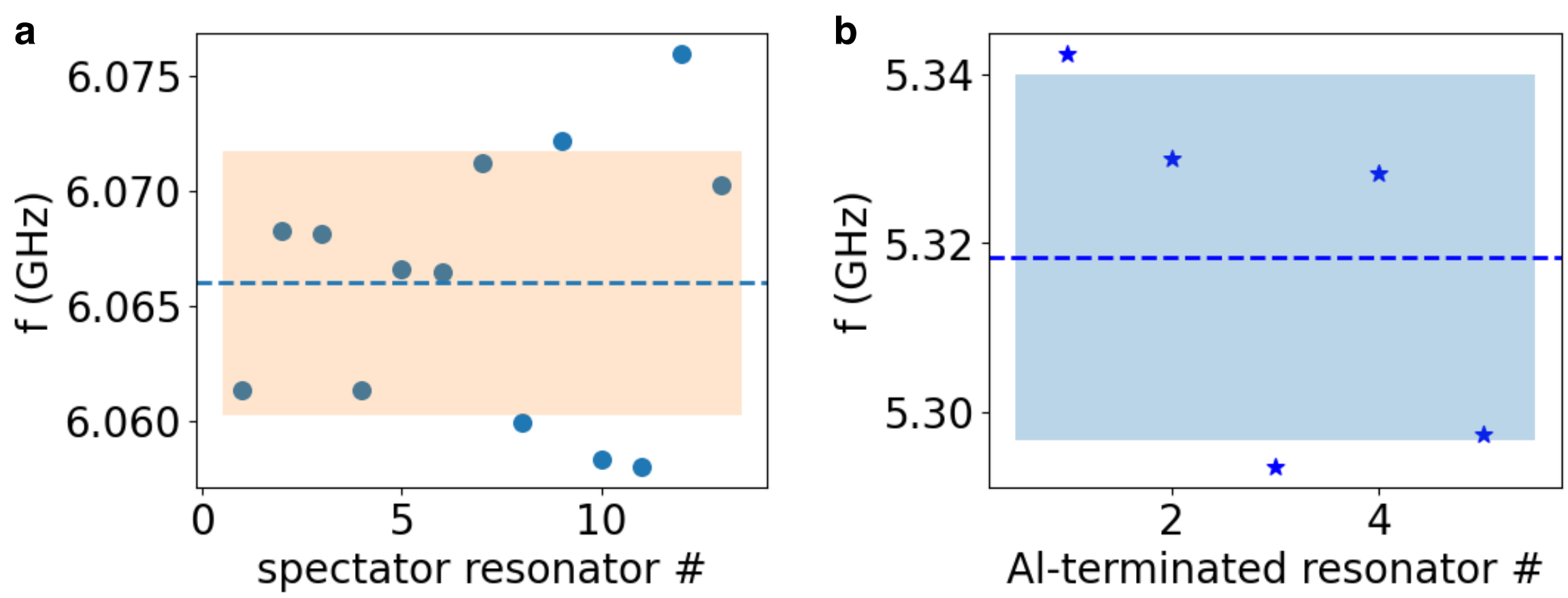

Figure 1: **Experimentally measured resonant frequencies of a, 12 *spectator* resonators exhibit a mean resonant frequency of 6.066 GHz (dashed line), with a standard deviation of 5.74 MHz, and b, 5 Al-terminated *experimental* resonators with nominally identical geometry as device D3. The distribution has a mean resonant frequency of 5.318 GHz (dashed line) with a standard deviation of 21.65 MHz.**

These measurements establish the expected device-to-device variation in resonance frequency for nominally identical designs. We note that for devices with a substantial kinetic inductance contribution (D1–D10), the induced frequency shift is much larger than the fabrication-induced spread, enabling the reliable extraction of kinetic inductance. In contrast, for devices with smaller kinetic inductance contribution (e.g., D11 and D12), the corresponding frequency shifts become comparable to the intrinsic fabrication variance, limiting the precision of the extracted values.

3. If not via experiments, do their simulations across various designs provide an insight into the variance in resonance frequency?

To quantify the variation in the control resonator frequency, we fabricated five Al-terminated control

resonators with nominally identical geometry to device D3. Across these five devices, we obtain a mean resonance frequency of 5.318 GHz with a standard deviation of 21.65 MHz (relative variation $\sim 0.41\%$). We note that for devices with a substantial kinetic inductance contribution (D1–D10), the induced frequency shift is much larger than the fabrication-induced spread, enabling the reliable extraction of kinetic inductance. In contrast, for devices with smaller kinetic inductance contribution (e.g., D11 and D12), the corresponding frequency shifts become comparable to the intrinsic fabrication variance, limiting the precision of the extracted values.

4. What is the impact of the contacts, both Al-contacts to $NbSe_2$ and the bridging contacts between Al, on the resonance frequency?

The contact resistances from Al-contacts to $NbSe_2$ and the bridging contacts between Al will act as a series resistance to the $\lambda/4$ resonator. A series resistance would decrease the internal quality factor $Q_{\rm i}$ for the $NbSe_2$-terminated resonator, but does not affect the resonant frequency. This is also supported by our simulation shown in Fig. 2. We use the SONNET finite-element solver to determine the resonant frequency of the coplanar waveguide resonator. We model the Al film as a two-dimensional perfect electrical conductor on a 350 μm silicon (Si) substrate, with 500 μm of vacuum above the film, and calculate the S-parameters at the microwave ports.

The lumped-element model for a $\lambda/4$ resonator terminated to ground with a resistance R = 0.0, 0.1, 0.2, 0.3, 0.4, and 0.5 Ω representing the contact resistance in series with the superconducting device. The simulation illustrates that while the resonant frequency remains essentially unchanged, the internal quality factor $Q_{\rm i}$ is sensitive to the series resistance, as it decreases rapidly with even a small resistance of 0.5 Ω.

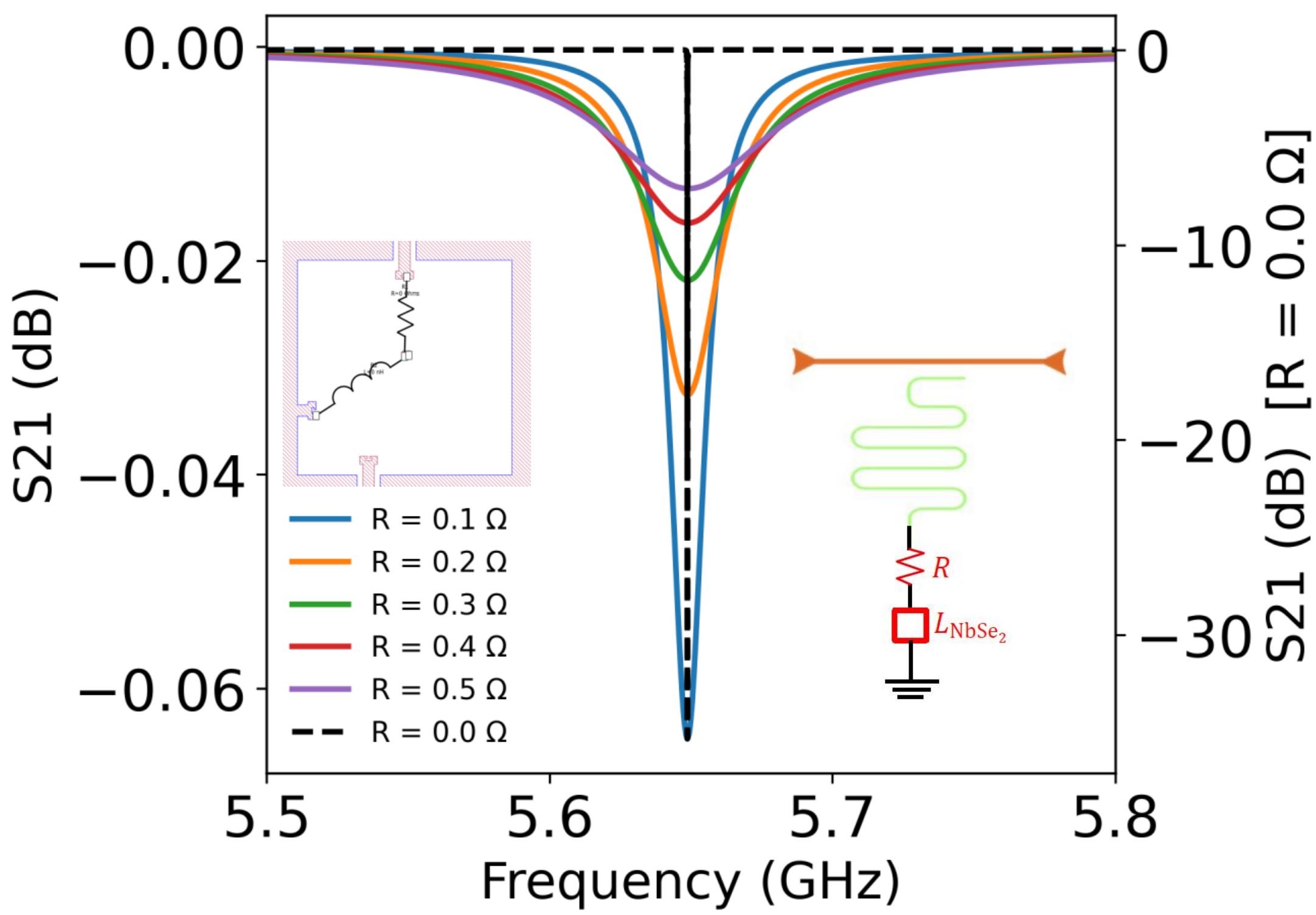

Figure 2: **Microwave simulation of superconducting $\lambda/4$ resonators terminated with a series resistance R. The resistance R has been swept from 0 Ω to 0.1 Ω in the lump-element model (shown in the insets). The resonance frequency shows a negligible change with R.**

5. What is the impact of adding dc contacts and LC filters on the resonance frequency?

   We thank the reviewer for pointing out a feature for further discussion. To address this, we are considering two scenarios:

   - **Presence of filters without connections**: We simulated $\lambda/2$ experimental resonators both with and without LC filters on the chip, observing no impact on resonator frequency. Figure 3a shows the microwave simulations with up to three filters added, demonstrating that the change in resonant frequency is negligible. The chips listed in Table 1 (main text) have on-chip filters, and only devices D6 and D10 had one DC electrode connected to the sample.
   - **Filter connected to the resonator**: We simulated the $\lambda/4$ experimental resonator with and without a DC line and filters connected to its terminated end (Fig. 3b). We observed a frequency shift of 4.1 MHz between the two configurations. This shift likely originates from the change

in impedance caused by the addition of the DC line and the LC filter. We emphasize that this shift has been accounted for by utilizing the all-aluminum control resonator (Device ID D6 and D10) with an identical configuration to the sample resonator, which effectively accounts for these geometric effects. In addition, the shift caused by adding DC electrodes and LC filters is smaller than the shift caused by $NbSe_2$ (ranging from 20 MHz to 1.68 GHz).

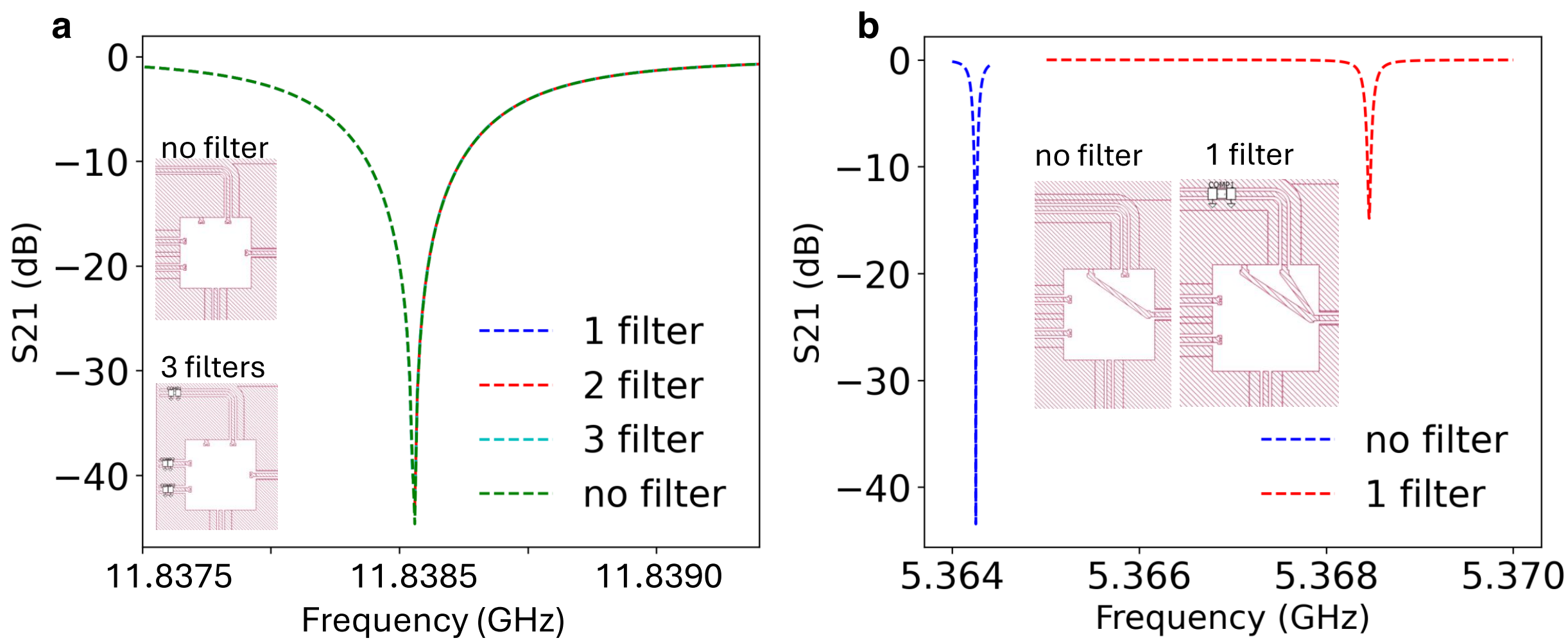

Figure 3: **Microwave simulations of superconducting resonators. a, $\lambda/2$ resonators with on-chip filters. b, $\lambda/4$ resonators with and without an electrode connection through a filter.**

6. The authors apply a dc current which increases the kinetic inductance quadratically as expected. Can the authors measure the critical current via dc measurements, use this to estimate I^*. And extract the kinetic inductance from the fitting? How does it match with the kinetic inductance estimated from the frequency shift?

   We thank the reviewer for this insightful suggestion. While DC transport measurements were not performed on this specific device due to the device's 2-probe configuration and the series Al elements (LC filter), we did estimate the critical current of another device of 8 nm thickness (close to the device reported in Fig 3 main text) in Fig. S1 (e-f), which yielded a critical current density $J_c = 2 \times 10^{10}$ A/m^2. This corresponds to a critical current of approximately $I_c \approx 0.7$ mA for our device dimensions. This value is comparable to the characteristic current $I^* \approx$ (0.4 mA) extracted from the microwave measurement, indicating reasonable agreement (within the same order of magnitude). This agreement between the DC and microwave results supports the conclusions that the observed non-linearity arises from the intrinsic kinetic inductance of the $NbSe_2$ flake.

   Line 176: It is not clear why the authors state that the '...quadratic dependence on the DC bias current

(Fig. 3f), [is] consistent with the anisotropic nodeless pairing of $NbSe_2$ reported in previous studies [43, 60].' This holds true in general in the Ginzburg-Landau framework as the authors state just before and anisotropic gap should have no impact on this dependence. If the authors' emphasis is on a nodeless gap, please clarify for the reader.

We thank the reviewer for pointing out this lack of clarity. The quadratic dependence of the resonance frequency shift on the applied dc bias current follows directly from the Ginzburg–Landau description of the nonlinear kinetic inductance. Within this framework, the superfluid density decreases quadratically with current, leading to a corresponding quadratic increase in kinetic inductance and hence a quadratic shift of the resonance frequency.

Our intention in referring to the anisotropic nodeless pairing reported for $NbSe_2$ in Refs. [43,60] was not to suggest that the anisotropy of the superconducting gap is responsible for this quadratic dependence. Rather, the key point is that $NbSe_2$ is known to host a fully gapped (nodeless) superconducting state, albeit with moderate gap anisotropy. In such systems, the nonlinear kinetic inductance is expected to follow the standard Ginzburg–Landau behavior at low currents, producing the observed quadratic dependence. To avoid confusion, we have revised the text (main text: line 177-178) to clarify that the quadratic current dependence arises from the general Ginzburg–Landau description of superconducting kinetic inductance, and that our observation is consistent with the expected behavior of a fully gapped superconducting state in $NbSe_2$, rather than being a direct signature of gap anisotropy.

The revised text reads: "We observe that both the resonant frequency and the kinetic inductance per square exhibit a quadratic dependence on the DC bias current (Fig. 3f), consistent with the fully gapped pairing of $NbSe_2$ reported in previous studies [46, 63]"

Line 126: The authors write 'To achieve a highly transparent, superconducting contact, we make [...] followed by in-situ argon ion milling and aluminum deposition'. And they list the milling parameters in Table S1. Furthermore, they write. Line 502: 'We chose the optimum ion milling time of 105 seconds...' However, from the information they provide it is not clear if milling has a systematic impact on contact resistance. Except for a milling time of 80 s, where the contact resistance is large, other milling times seem to have no impact. It is not clear why 105 s is the optimal time.

The authors should clarify the impact of no Ar milling on contact resistance? Or if they tried much longer milling times?

We thank the reviewer for pointing out this lack of clarity. We agree that the term "optimum" is misleading, as the contact resistance (R_c) remains consistently low across a range of milling times from

60s to 120s.

Defining the Choice of 105 s: The duration of 105 s was chosen as a standardized, reliable condition within the stable regime observed in Table S1. While 60 s and 90 s also yielded low R_c, 105 s was selected to ensure complete removal of any potential polymer residues or atmospheric contaminants across the entire contact area before Aluminum deposition, providing high reproducibility across multiple device batches.

Impact of "No Milling": Our experiments, consistent with a previous study by [4], show that omitting the Ar milling step entirely resulted in inconsistent contacts with significantly higher resistances. This is attributed to the rapid formation of a native oxide layer on $NbSe_2$. The data in Table S1 demonstrates that milling is necessary to "break" through this oxide layer.

Longer Milling Times: We tested ion-milling times up to 120 s, beyond 90 s, we observed no further reduction in R_c (staying at 0.01 Ω/μm). We did not pursue significantly longer times to avoid over-etching the thin 2D flake ($d \ll 10$ nm), as excessive ion bombardment can degrade the sample and suppress the local T_c.

We have revised the text (Line 126 and Line 516) to replace "optimum" with "chosen reproducible condition" to better reflect that 105 s is within a broad, functional plateau of low contact resistance.

The revised text now reads: "We present DC transport measurements to characterize the contact resistance as a function of ion milling time, with the results summarized in Table S1. We selected 105 s for the ion milling time, which we demonstrated to provide a reproducible and low contact resistance."

7. The text unfortunately doesn't cite some key references. One crucial example is [6a] M. Kriedel et al. PhysRevResearch 6, 043245 (2024) where the authors measured the kinetic inductance of $NbSe_2$, using the same techniques - integrating it into a microwave resonator. Furthermore, often the reader gets an impression that these are the first microwave measurements in materials other than in amorphous superconducting thin films.

   Line 59: 'Recent efforts toward this goal have focused on implementing superinductors using amorphous superconducting thin films'

   Line 187: 'Notably, $NbSe_2$, particularly in the few-monolayer regime, exhibits one of the highest sheet kinetic inductances reported at comparable thicknesses, surpassing those of many conventional high-impedance superconductors.'

   Line 241: '...reaches 1.2 nH/sq at monolayer thickness — among the highest reported for supercon-

ductors within this thickness regime.'

This is not true. The authors own works have measured this in graphene-based superconductors. And other van der Waals superconductors have been probed. [6b] Tanaka, M. et al. Nature 638, 99–105 (2025). [6c] Banerjee, A. et al. Nature 638, 93–98 (2025). [6d] Jin, H. et al. Nano Lett. 2025, 25, 8, 3191–3198 (2025). [6e] Jha, R. et al. Phys. Rev. Lett. 134, 216001 (2025). [6f] Tian, H. et al. Nature 614, 440–444 (2023).

I suggest that the authors provide the reader with a comprehensive overview of the relevant references.

We thank the reviewer for mentioning these important prior demonstrations, and we fully agree they should be acknowledged. We have cited them now on page 2 line 50 (main text).

We have also revised the language in the main text to distinguish the intrinsic superconductors, which are listed in fig 4b, and the voltage-induced superconductor, including MATBG or MATTG.

The revised text reads: "Voltage-induced flat-band superconductors, such as magic-angle twisted bilayer [4, 66] and trilayer graphene [5, 67], exhibit a wide range of inductances depending on bias voltages and are therefore not included in Fig. 4b."

8. How is the error in $L_{k,sq}$, error bars in Fig. 4a estimated?

   The error bars in Fig. 4a reflect the geometric uncertainty in the effective number of squares. Because the $NbSe_2$ flakes deviate slightly from an ideal rectangulary geometry, we estimate the uncertainty in the number of squares by comparing the effective rectangular area between the electrodes to the outer flake envelope extracted from microscopy images. The corresponding fractional uncertainty is propagated directly to the error bars.

   In addition, we incorporate the fit error from the resonance frequency extraction obtained via circle fitting of the S_{21} data [5, 6]. This contribution is included for both the $NbSe_2$-terminated and the control resonators.

   (Error bars are updated in Fig. 4a)

9. There's a typo in equation 27, in the supplementary information.

   The typo is corrected.

References

1. Dvir, T. *et al.* Spectroscopy of bulk and few-layer superconducting $NbSe_2$ with van der Waals tunnel junctions. *Nature Communications* **9,** 598 (2018).
2. Khestanova, E. *et al.* Unusual suppression of the superconducting energy gap and critical temperature in atomically thin $NbSe_2$. *Nano Letters* **18,** 2623–2629 (2018).
3. Cao, Y. *et al.* Quality heterostructures from two-dimensional crystals unstable in air by their assembly in inert atmosphere. *Nano Letters* **15,** 4914–4921 (2015).
4. Sinko, M. R. *et al.* Superconducting contact and quantum interference between two-dimensional van der Waals and three-dimensional conventional superconductors. *Physical Review Materials* **5,** 014001 (2021).
5. Probst, S., Song, F., Bushev, P. A., Ustinov, A. V. & Weides, M. Efficient and robust analysis of complex scattering data under noise in microwave resonators. *Review of Scientific Instruments* **86** (2015).
6. Baity, P. G. *et al.* Circle fit optimization for resonator quality factor measurements: Point redistribution for maximal accuracy. *Physical Review Research* **6,** 013329 (2024).

Round 2

Reviewer 1

The authors had addressed my question. I support its publication.

We thank the reviewer for the very positive assessment of our manuscript. We are pleased that our revisions have addressed the reviewer's comments, and we sincerely appreciate the reviewer's recommendation for publication in Nature Communications.

Reviewer 2

The authors have provided sufficient information to support their claims. I support the publication of the article in Nature Communications.

We thank the reviewer for the very positive assessment of our manuscript. We are pleased that our revisions have addressed the reviewer's comments, and we sincerely appreciate the reviewer's recommendation for publication in Nature Communications.
